# Chemotherapeutic Activity of Imidazolium-Supported Pd(II) *o*-Vanillylidene Diaminocyclohexane Complexes Immobilized in Nanolipid as Inhibitors for HER2/neu and FGFR2/FGF2 Axis Overexpression in Breast Cancer Cells

**DOI:** 10.3390/ph16121711

**Published:** 2023-12-11

**Authors:** Aeshah A. Awaji, Moustafa A. Rizk, Raiedhah A. Alsaiari, Norah F. Alqahtani, Fatima A. Al-Qadri, Ali S. Alkorbi, Hani S. Hafez, Reda F. M. Elshaarawy

**Affiliations:** 1 Department of Biology, Faculty of Science, University College in Taymaa, University of Tabuk, Tabuk 71491, Saudi Arabia; aawaji@ut.edu.sa; 2 Department of Chemistry, Faculty of Science and Arts at Sharurah, Najran University, Sharurah 68342, Saudi Arabia or moustafarizk@science.suez.edu.eg (M.A.R.); raalsayari@nu.edu.sa (R.A.A.); fatimaalqadri@gmail.com (F.A.A.-Q.); assalem@nu.edu.sa (A.S.A.); 3 Department of Chemistry, College of Science, University of Jeddah, Jeddah 21589, Saudi Arabia; nfalqahtani@uj.edu.sa; 4 Zoology Department, Faculty of Science, Suez University, Suez 43533, Egypt; 5 Department of Chemistry, Faculty of Science, Suez University, Suez 43533, Egypt; 6 Institut für Anorganische Chemie und Strukturchemie, Heinrich-Heine Universität Düsseldorf, 40204 Düsseldorf, Germany

**Keywords:** imidazolium–vanillylidene ligands, Pd(II) complexes-enacapsulated nanolipids, cytotoxicity, HER2/neu, FGFR2/FGF2, apoptosis

## Abstract

Two bis-(imidazolium–vanillylidene)-(*R*,*R*)-diaminocyclohexane ligands (H_2_(VAN)_2_dach, H_2_L_1,2_) and their Pd(II) complexes (PdL_1_ and PdL_2_) were successfully synthesized and structurally characterized using microanalytical and spectral methods. Subsequently, to target the development of new effective and safe anti-breast cancer chemotherapeutic agents, these complexes were encapsulated by lipid nanoparticles (LNPs) to formulate (PdL_1_LNP and PdL_2_LNP), which are physicochemically and morphologically characterized. PdL_1_LNP and PdL_2_LNP significantly cause DNA fragmentation in MCF-7 cells, while trastuzumab has a 10% damaging activity. Additionally, the encapsulated Pd_1,2_LNPs complexes activated the apoptotic mechanisms through the upregulated P53 with *p* < 0.001 and *p* < 0.05, respectively. The apoptotic activity may be triggered through the activity mechanism of the Pd_1,2_LNPs in the inhibitory actions against the FGFR2/FGF2 axis on the gene level with *p* < 0.001 and the Her2/neu with *p* < 0.05 and *p* < 0.01. All these aspects have triggered the activity of the PdL_1_LNP and PdL_2_LNP to downregulate TGFβ1 by *p* < 0.01 for both complexes. In conclusion, LNP-encapsulated Pd(II) complexes can be employed as anti-cancer drugs with additional benefits in regulating the signal mechanisms of the apoptotic mechanisms among breast cancer cells with chemotherapeutic-safe actions.

## 1. Introduction

The overexpression of the human epidermal receptor 2 (HER2) gene is a prominent feature in various cancers, particularly breast cancer, and is associated with aggressive tumor growth, poor prognosis, and an increased risk of metastasis [1]. Overexpressing HER2 leads to increased activation of PI3K/AKT and MAPK downstream signaling pathways that trigger uncontrolled cancer cell growth and progression [2]. Designing effective HER2 overexpression inhibitors is crucial for targeted therapies, like trastuzumab, targeting the HER2 receptor and inhibiting its signaling pathway [3]. Trastuzumab has been shown to improve survival outcomes and reduce the risk of disease recurrence in HER2-positive breast cancer patients [4]. Advancements in targeted therapies require further research to control HER2 overexpression.

Platinum-based drugs like cisplatin and carboplatin show efficacy against tumors, but side effects and resistance limit their widespread use [5]. On the other hand, Pd(II) complexes have shown promise as potential alternatives to platinum-based drugs [6]. Pd(II) complexes target DNA, forming covalent adducts, and exhibit higher selectivity towards cancer cells due to structural differences, reducing toxicity risk [7]. Interestingly, padeliporfin represents a significant milestone in cancer therapy as the first Pd-based clinical photodynamic drug [8]. Despite the promising characteristics of Pd(II) complexes, further research is required to fully overcome the significant limitations of Pd(II) complexes as anticancer agents with solubility limitations [9]. In this context, various strategies have been used to improve the selectivity and efficacy of palladium complex-based cancer therapies. One such approach involves the design of novel ligands that can selectively target cancer cells while sparing healthy cells. By incorporating specific functional groups into the ligands, researchers have been able to achieve targeted delivery of the palladium complexes to cancerous tissues. Additionally, the development of nanoscale drug delivery systems has enabled the precise delivery of palladium complexes to tumor sites, thereby minimizing systemic toxicity and improving therapeutic outcomes [9]. Therefore, multifunctional delivery systems such as nanocarriers for Pd(II) complexes are needed to improve biomedical properties [10]. Notably, the passive targeting ability of nanoparticles with a diameter of 20–200 nm on solid tumor tissues has gained widespread acceptance [11,12,13]. The targeted approach enhances cancer treatment efficacy by delivering therapeutic agents directly to tumor sites through passive targeting, enhancing permeability and retention effects in nanostructures [14,15], micelles [16,17], liposomes [17,18,19], and nanoemulsions [20]. Additionally, FGF2/FGFR signaling changes are prevalent in various tumor types, with FGF2 being overexpressed in advanced malignant tumors, FGFR2 inhibitors playing a crucial role in tumor growth and progression, and overcoming anticancer drug resistance through novel extracellular inhibitors [21].

Lipid nanoparticles (LNPs) are promising delivery systems for metal complexes due to their biocompatibility, stability, controlled release, efficient encapsulation, targeted delivery, and potential for improved therapeutic efficacy and reduced toxicity. They protect complexes from degradation, enhance solubility, and can be easily modified for targeted delivery [22]. These key factors make LNPs an attractive option for delivering metal complexes in medicinal and pharmaceutical applications [23].

Notably, Metallo-Schiff base complexes, particularly vanillylidene (VAN)-based, have gained significant attention as potential chemical nucleases in cancer treatment. These complexes have demonstrated promising DNA cleavage activity through various mechanisms, such as the generation of reactive oxygen species (ROS) that induce oxidative damage to the DNA backbone and the direct coordination of the metal center with DNA, which can distort the DNA structure and facilitate DNA cleavage [24,25]. Pd(II) Schiff base complexes inhibit breast cancer cell growth and spread, exhibiting anti-angiogenic properties and inhibiting blood vessel formation for nutrients [26,27], offering new treatment options for patients who have developed resistance to standard therapies [28,29].

These aforesaid outstanding facts, coupled with our continuous interest in exploring new safe chemotherapeutic agents, motivated us to design and develop two imidazolium-supported vanillylidene ligands (*R,R*-H_2_(VAN)_2_dach(*^n^*BuIm^+^-X^–^)_2_ ligands (H_2_L_1,2_)) and their Pd(II)-(VAN)_2_dach complexes ((VAN)_2_dach = *N,N*′-bis-(vanillylidene)-*R,R*-1,2- diaminocyclohexane). These complexes will then be loaded on LNPs to formulate PdL_1_LPN and PdL_2_LPN for human breast cancer therapy. Furthermore, this study aims to examine the chemotherapeutic efficacy of novel formulations as inhibitors of the Her2/neu pathway and their ability to target the FGFR2/FGF2 axis mechanisms and enhance the apoptotic activity in cancer cells.

## 2. Results

### 2.1. Chemistry of Synthesis

**Scheme 1:**
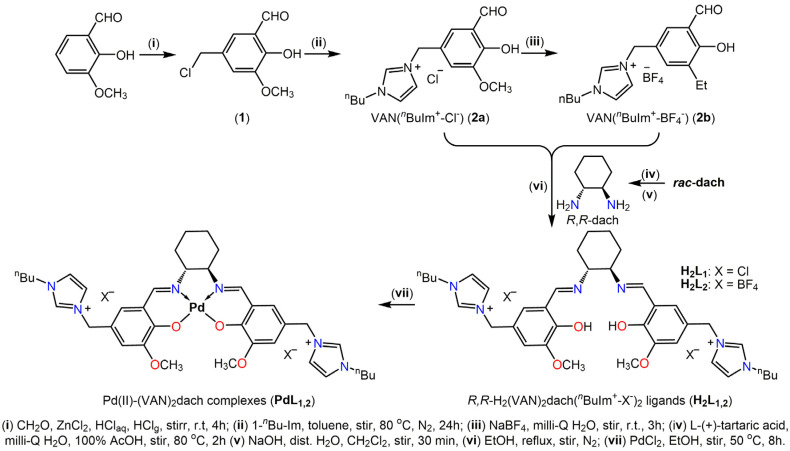
Step by step synthesis of VAN(*^n^*BuIm^+^-X^-^) (2a,b), imidazolium–vanillylidene ligands (*R,R*-H_2_(VAN)_2_dach(*^n^*BuIm^+^-X^-^)_2_ (H_2_L_1,2_), and Pd(II)-(VAN)_2_dach (PdL_1,2_) complexes.

The ligands used in this study, imidazolium–vanillylidene ligands (*R,R*-H_2_(VAN)_2_dach(*^n^*BuIm^+^-X^–^)_2_, (H_2_L_1,2_)), were synthesized through the Schiff base condensation reactions between the pre-prepared vanillyl–butyl–imidazolium ionic liquids (VAN(*^n^*BuIm^+^-X^–^), 2a,b) and *R,R*-dach in a 2:1 molar ratio. The reactions were carried out in an ethanolic solution using the Schlenck method under a N_2_ atmosphere (see Scheme 1). Initially, VAN(*^n^*BuIm^+^-X^–^) (2a,b) and *R,R*-dach were synthesized using protocols modified from our previous work [30] and then subjected to the Schiff base condensation reactions. After that, the Pd(II)-(VAN)_2_dach (PdL_1,2_) complexes were prepared via the reaction of the new ligands with PdCl_2_ in an ethanolic medium containing conc HCl. Finally, the microemulsion process was employed to load the PdL_1,2_ complexes into the LNPs network, targeting the preparation of PdL_1,2_-loaded LNPs (PdL_1,2_LNPs).

### 2.2. Characterization of New Materials

Acceptable yields were obtained for the free ligands, their Pd(II) complexes, and PdL1,2-loaded LNPs (PdL_1,2_LNPs). The structural characterization of the compounds was conducted using microanalytical and spectral analysis techniques, including Fourier-transform infrared spectroscopy (FTIR), ultraviolet–visible spectroscopy (UV–Vis), nuclear magnetic resonance spectroscopy (^1^H, ^13^C, ^19^F, ^11^B NMR), and electrospray ionization mass spectrometry (ESI-MS). Moreover, PdL_1,2_LNPs were analyzed for their morphology using TEM analysis.

#### 2.2.1. Physicochemical Characterization 

Elemental CHN analyses performed on both free ligands and their Pd(II) complexes were found to be satisfactory (see experimental Section 4.2), and the results agreed with the hypothesized structural formulas for the ligands and complexes described in the Experimental section. In addition, the sequential loss of anionic species (Cl^−^ and BF_4_^−^) from the native H_2_L_1,2_ and PdL_1,2_ are reflected in the electrospray ionization mass spectrometry (ESI-MS) of them in a positive mode (+ve ESI-MS) as dominating peaks for the singly and doubly charged cations.

The initial observation of the ^1^H NMR spectra of the new imidazolium–vanillylidene ligands (Figures S1 and S2, ESM†) appears complex due to the presence of two distinct signal sets. This complexity can be attributed to the coexistence of two tautomeric pairs, bis-(enolimine) and the ionic imine-zwitterionic form, each with varying populations. The predominance of the bis-enolimine tautomeric form over the imine-zwitterionic form can be inferred from the observed ^1^H NMR spectral features of H_2_L_1_/H_2_L_2_. These features include two distinct singlets at 10.59/13.80 and 10.53/9.20 ppm, which can be attributed to the resonance of the phenolic OH group. These singlets are characteristic of the bis-enolimine tautomer. The presence of a weak doublet at 8.92 ppm (J = 7.2 Hz) suggests the resonance of an iminic proton, which is commonly observed in ethylenic protons of zwitterionic fragments. Additional proof that novel ligands were successfully formed and that two tautomeric forms exist in the deuterated solutions of these ligands is provided using ^13^C NMR spectroscopy (Figures S3 and S4, ESM†). ^11^B-NMR and ^19^F-NMR spectroscopy were used to probe the counter-anion structures of the H_2_L_2_ ligand. The presence of BF_4_ as a counter anion for H_2_L_2_ is confirmed by the appearance of two singlets at chemical shifts of −1.30 and −148.67 ppm in the ^11^B NMR and ^19^F NMR spectra of H_2_L_2_ (Figures S5 and S6, ESM†).

**Figure 1:**
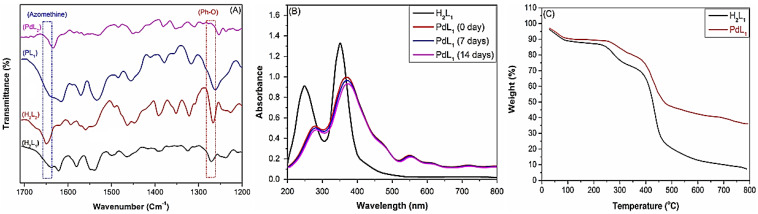
(**A**) Selected IR spectral area (1710–1200 cm^−1^) demonstrates how coordination with Pd(II) ions modifies the azomethine and phenolic peaks in the H_2_(VAN)_2_dach(^n^BuIm^+^-X^–^)_2_ (**H_2_L_1,2_**) spectra. (**B**) UV–Vis spectra of H_2_L_1_ and its Pd(II) complex, Pd(VAN)_2_dach(^n^BuIm^+^-Cl^–^)_2_ (**PdL_1_**) (0.001 M) in a DMSO:PBS solution (pH = 7.4) at 25 °C. (**C**) TGA curves of H_2_L_1_ and PdL_1_.

The FTIR spectral analysis of the new imidazolium–vanillylidene ligands and their Pd(II) complexes (Figure 1A) and (Figures S7–S10, ESM†) provides strong evidence for their successful formation. The distinct IR signatures observed, coupled with the tentative assignments, highlight the presence of key functional groups in these compounds. The IR spectra of the parent ligands exhibit prominent peaks at approximately 3438 ± 1, 1635, 1274 ± 2, and 752 cm^−1^, which correspond to the stretching vibrations of phenolic-OH, azomethine H-C=N, Ph-O, and imidazolium groups, respectively. These peaks are distinctive for their H_2_(VAN)_2_dach(^n^BuIm^+^-X^–^)_2_ (H_2_L_1,2_) structural patterns [31]. These stretching vibration bands might be employed as meaningful spectral Pd(II)-(VAN)_2_dach binding indicators since they were either absent from or changed their sites or intensities in the spectra of Pd(II)-(VAN)_2_dach complexes. For instance, the deprotonation of the phenolic-OH group and coordination of the phenolate oxygen to the Pd(II) ion are evidenced by the disappearance of the phenolic-OH band and a decrease in the intensity of the phenyl-O stretch as well as its negative shift by 8 ± 1 cm^−1^. Meanwhile, as a result of the azomethine nitrogen being bound to the Pd(II) ion, the spectral position of the characteristic azomethine (H-C=N) peak for the free ligands is negatively shifted by 17–18 cm^−1^ in the spectra of complexes. The results demonstrate that the new ligands act as tetradentate dianionic ligands, forming stable complexes with Pd(II) ion through N,N,O,O binding sites (see Scheme 1) [32].

The UV spectrum of the H_2_(VAN)_2_dach(^n^BuIm^+^-Cl^–^)_2_ (H_2_L_1_) ligand (Figure 1B) shows two prominent absorption peaks at around 249–251 nm and 351–352 nm, which were attributed to the π→π* and n→π* transitions, respectively, due to the phenyl ring and C=N meioty. Noteworthy, upon complexation with Pd(II), the absorption peaks in the UV spectra of the ligand shifted to longer wavelengths, and their intensities decreased, indicating a redshift and a hypochromisim in the electronic transitions. Specifically, the absorption peak at around 250 nm shifted to 273 nm, while the peak at around 351 nm shifted to 367 nm. This redshift in the absorption peaks can be attributed to the coordination of the Pd(II) ion with the imidazolium–vanillylidene ligand, which results in a change in the electronic environment around the ligand. Interestingly, PdL_1,2_(II) complexes exhibit characteristic UV absorption bands at around 550 nm resulting from ligand-to-metal charge transfer (LMCT) and metal-to-ligand charge transfer (MLCT) transitions typically for square planar geometries [33]. Furthermore, the intensity of these peaks can provide valuable information about the concentration and stability of the complex. By monitoring changes in the intensity of this peak over time, researchers can gain insights into the stability of the complex. Notably, Pd(II) complexes are stable under physiological conditions (pH 7.4), as shown by the UV–Vis spectrum (Figure 1B), which showed no significant change in the spectrum of complex solution after 2 weeks of storage at ambient conditions.

Thermogravimetry analysis (TGA) was employed to examine the thermal stability of the new ligands and their Pd(II) complexes. The results of the TGA analysis revealed that the Pd(II) complex (PdL1) exhibited higher thermal stability compared to the free ligand (Figure 1C), with no significant weight loss observed up to 270 °C. This could be attributed to the coordination between the Pd(II) ion and the ligand, which strengthens the overall stability of the complex. These findings indicate that the Pd(II)-(VAN)_2_dach complexes can withstand high temperatures, making them a promising candidate for various applications that require thermal stability.

**Figure 2:**
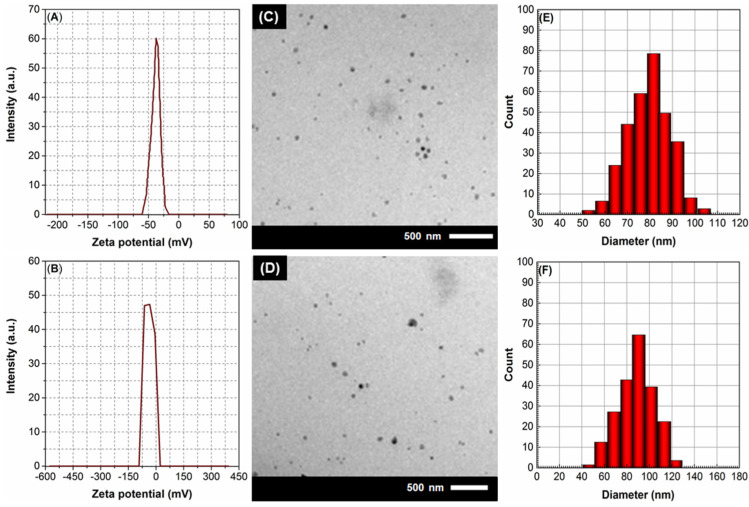
(**A**,**B**) Zeta potential, (**C**,**D**) TEM nanographs, and (**D**–**F**) particle size distribution (PSD) of the PdL_1_LNPs and PdL_2_LNPs, respectively.

Zeta potential, which is the net electric charge on the surface of nanoparticles, plays a significant role in determining their stability and behavior in biological systems. Good stability over time in storage is indicated by a zeta potential greater than |30 mV*|* [34]. The LNPs loaded with PdL_1_ and PdL_2_ (PdL_1_LNPs and PdL_2_LNPs) exhibited zeta potentials of −37.53 ± 3.98 mV and −34.41 ± 3.81 mV (Figure 2A,B). The increased negative zeta potential can have important implications for the stability and colloidal behavior of these nanoparticles, as it enhances their electrostatic repulsion, preventing aggregation and promoting their dispersion in aqueous solutions.

#### 2.2.2. Morphological Characterization 

The morphological properties of the new Pd(II) complexes-loaded LNPs are revealed using TEM imaging. TEM imaging (Figure 2C,D) revealed that the PdL_1_LNPs and PdL_2_LNPs exhibited a spherical morphology with a narrow size distribution. The TEM images also indicated that the nanoparticles possessed a homogeneous and compact internal structure, suggesting the successful entrapment of the Pd(II) complex within the lipid matrix. With this consistency in size, drug loading and release characteristics may be optimized, which is crucial for the use of LNPs in drug delivery systems (DDSs). By measuring the diameters of 67 individual nanoparticles in each TEM picture, the average diameters of PdL_1_LNPs and PdL_2_LNPs were found to be 69.85 ± 3.1 nm and 73.91 ± 2.8 nm, respectively.

The dynamic light scattering (DLS) technique offers a non-invasive and efficient means of characterizing the size distribution and stability of nanoparticles, allowing researchers to optimize their formulation and tailor their properties for specific applications [35]. By revealing crucial information regarding the particle size, polydispersity (PDI), and aggregation state, DLS analysis helps to ensure the successful development and utilization of Pd(II) complexes-loaded lipid nanoparticles in various fields, including drug delivery and diagnostics. The DLS analysis results (Figure 2E,F) showed that both PdL_1_LNPs and PdL_2_LNPs exhibited unimodal size distributions, indicating a narrow size range of nanoparticles. The unimodal size distribution of nano gels is essential for their efficacy as DDSs, as it guarantees uniform drug encapsulation and release kinetics [36]. It is worth noting that the mean hydrodynamic diameter of PdL_1_LNPs was measured to be 81.53 ± 2.6 nm, while for PdL_2_LNPs, it was measured to be 90.50 ± 3.3 nm. Furthermore, the PDI values for the PdL_1_LNPs and PdL_2_LNPs were 0.17 ± 0.02 to 0.24 ± 0.05. The low PDI value is desirable as it indicates a homogenous distribution of Pd(II) complexes-loaded LNP sizes. This is crucial for the efficient delivery of Pd(II) complexes, as a low PDI value ensures uniform encapsulation and release of the complex [37].

#### 2.2.3. Pharmacological Characterization 

The entrapment efficiency (EE) parameter plays a significant role in determining the therapeutic efficacy and safety of the drug delivery system. As highlighted by Marquele-Oliveira et al., a higher entrapment efficiency ensures that a greater proportion of the drug is available for release at the target site, leading to enhanced therapeutic outcomes. On the other hand, a higher loading capacity allows for the delivery of a greater amount of drug, which is particularly important when dealing with drugs that have a low therapeutic index or when a high drug dosage is required [38]. The efficiency of encapsulating Pd(II) complexes in PdL_1_LNPs was 81.29 ± 3.95%, while it was 88.97 ± 2.12% in PdL_2_LNPs. The higher EE of Pd(II) complexes in LNPs could be attributed to several factors. Firstly, the incorporation of the complex in the surfactant layer on the lipid carriers’ outer surface the microemulsion formation, where the complex can partition from the oil phase to the aqueous phase. However, as the O/W microemulsion is cooled, the solubility of the drug in the water phase decreases, causing the complex to re-partition to the lipid phase [36]. Moreover, the surfactant molecules form a protective layer around the complex, preventing its premature release and degradation. This allows for higher retention of the Pd(II) complex within the LNPs, leading to an increased EE. Secondly, the interaction between the Pd(II) complex and the lipid bilayer is increased within the lipid nanoparticles, promoting a higher entrapment efficiency. The Pd(II) complex, which is amphiphilic, tends to strongly interact with the lipid bilayer. This interaction promotes entrapment efficiency by facilitating the incorporation of the Ru complex into the lipid bilayer structure. This interaction further enhances the stability and integrity of the lipid vesicles, thereby improving the overall entrapment efficiency. On the other hand, the LC of Pd(II) complex in PdL_1_LNPs was 43.34 ± 2.76% while it was 47.85 ± 3.15% in PdL_2_LNPs.

**Figure 3:**
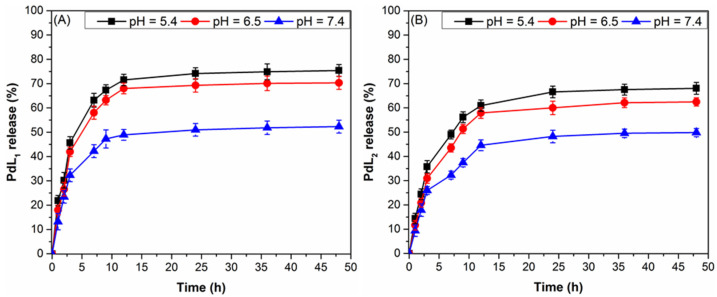
(**A**) PdL_1_ and (**B**) PdL_2_ release patterns from LNPs in vitro at 37 °C and in physiological and acidic environments (pH 7.4, pH 6.5, and pH 5.4).

Studying the in vitro release kinetics of the Pd(II) complex from lipid nanoparticles provides valuable insights into the controlled release of therapeutic agents. So, we tested the release style of the Pd(II) complex when the PdL_1,2_LNPs were exposed to 37 °C and three distinct pH levels. The complex-loaded LNPs were tested in three different buffer media at pH 7.4, 6.5, and 5.4. These values were selected because they mimic the pH of intestinal fluid, human mammary cancer (MCF-7) cells, and the tumor environment. The intestinal fluid is generally characterized by a pH range of 6.5 to 7.5, with 7.4 being the most representative of the physiological pH in the small intestine. This specific pH level is crucial for the successful development and evaluation of nanoparticles intended for oral drug delivery systems. By simulating the intestinal fluid at pH 7.4, researchers can effectively investigate the behavior and stability of complex-loaded LNPs in an environment that closely resembles the actual conditions of the gastrointestinal tract [39]. The selection of this pH value is justified by its ability to provide valuable insights into the fate of nanoparticles, including their dissolution, release, and absorption mechanisms. Therefore, the use of pH 7.4 as a simulated intestinal fluid offers a reliable platform for testing and optimizing complex-loaded LNPs, ultimately contributing to the advancement of oral drug delivery systems. Noteworthy, both types of complex release from LNPs have been found to follow an intriguing three-phase pattern, beginning with a rapid burst phase (within the first 7 h), extremely sluggish complex release (within the next 17 h), and then a plateau stage (Figure 3). Possibilities for improved complex delivery regulation and enhanced therapeutic effects have been suggested using this unique Pd(II) complex release pattern. It is interesting to note that the proportion of PdL_1_ released increased from 40.6% to 54.2% and then to 52.3% after 7, 24, and 48 h of incubation at pH 7.4 (Figure 3A). However, for the same pH and time intervals, the percentages of PdL_2_ released were 33.3%, 51.2%, and 49.1%, respectively (Figure 3B). The limited Pd(II) complex release observed at pH 7.4 can be attributed to the stability of the ionic interaction between the amphiphilic Pd(II) complex and the lipid bilayer under neutral conditions. In contrast, the LNPs’ capability to release both complexes is much improved when the pH is dropped to 5.4. Overall, the new LNPs exhibited pH-responsive behavior, with a higher drug release rate observed at acidic pH values compared to neutral pH. The accelerated drug release from the nanogel at acidic pH could be attributed to the protonation of the ionizable groups on the LNPs’ surface at low pH levels, which leads to a decrease in the crosslinking density of the polymer network and an increase in the swelling of the LNPs. Such changes in the structure and swelling of the LNPs facilitate the diffusion of a complex out of the LNPs, resulting in an enhanced release [36].

### 2.3. Pharmacology and Biological Activities

#### 2.3.1. In Vitro Cytotoxicity 

Based on the IC_50_ values (see Table 1), it can be observed that the lipid-encapsulated Pd(II) complexes (IC_50_ 1.93 ± 0.29 μg/mL for PdL_1_LNPs and 2.03 ± 0.21 μg/mL for PdL_2_LNPs) exhibited enhanced cytotoxicity in comparison to their non-encapsulated counterparts (IC_50_ 16.28 ± 0.56 μg/mL for PdL_1_ and 23.72 ± 1.25 μg/mL for PdL_2_) when targeting breast cancer cells (MCF-7). This can be attributed to the ability of lipid-encapsulated complexes to protect the therapeutic agents from degradation and enhance their stability and solubility. Furthermore, the lipid bilayers of these complexes provide a favorable environment for incorporating various drugs, enabling combination therapy for breast cancer treatment. The lipid-based delivery systems have also been shown to enhance drug accumulation in tumor tissues due to the enhanced permeability and retention effect [40]. Interestingly, the anti-breast cancer activity of PdL_1,2_LNPs outperforms that of clinical drugs cisplatin (CDDP) and trastuzumab (TRZ). Furthermore, the lipid nanoparticles-encapsulated complexes have demonstrated a remarkable advantage over clinical drugs in terms of their selectivity index (SI) values towards cancer cells compared to healthy ones. The findings revealed that PdL_1,2_LNPs exhibited higher selectivity indices (20.19 and 20.33) compared to clinical drugs (13.66 and 8.18). This suggests that the use of lipid nanoparticles as drug delivery systems can potentially enhance the therapeutic effectiveness of anticancer agents while minimizing their toxicity to normal cells. 

#### 2.3.2. Comet Assay

**Figure 4:**
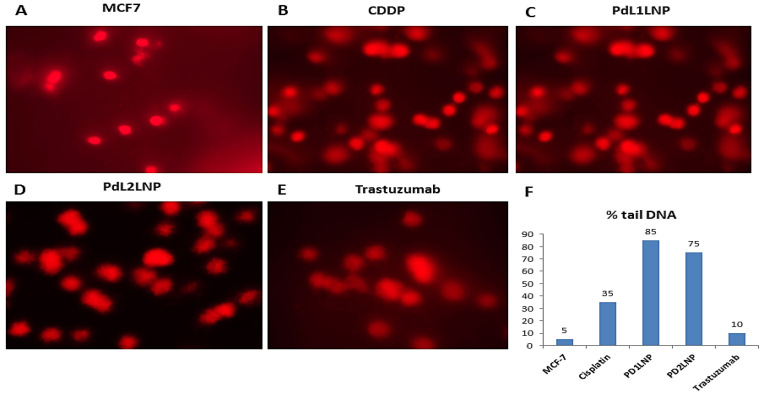
(**A**–**F**) Quantitative measure for 100 cells on a scale from 0 to 400.

The comet assay and percentages of the damaged DNA assay in the form of a tail indicate the quantity of genetic material dispersed in the broken pieces. More than 100 cells have been examined for each cellular structure of MCF-7 cells with and without variable drugs. The present data revealed the vigorous role of the PdL_1_LNP and PdL_2_LNP with ~85% and 75%, respectively, and 35% among cells treated with cisplatin in inducing significant DNA fragmentation in the MCF-7 cell lines, with a stumpy activity role for the trastuzumab with the damaging activity (Figure 4A–E). Data are presented in Figure 4F. The high percentage indicates the degree of damage, and most DNA is in the “tail”.

#### 2.3.3. Quantitative Real-Time (qPCR) Estimation for FGFR2 and FGF2

**Figure 5:**
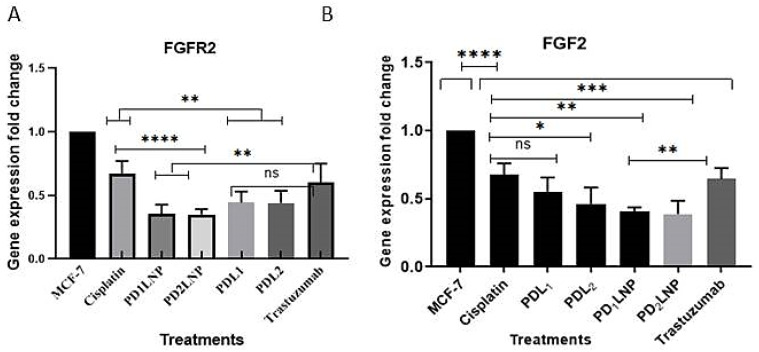
The effect of the lipid-encapsulated Pd(II) complexes in the form of PdL_1,2_LNPs (PD_1_LNP and PD_2_LNP) treatment on the quantitative gene expression of FGFR2 (**A**) and FGF2 (**B**) in MCF-7 carcinoma in comparison to cisplatin and trastuzumab. Mean ± SE. * *p* < 0.05, ** *p* < 0.01, *** *p* < 0.001. **** *p* < 0.0001, and ns, non-significance.

On one side, Pd(II) complexes (PdL_1_ and PdL_2_) revealed a significant decrease with *p* < 0.01 for the expression levels of FGFR2 (Figure 5A), while PDL1 expressed a non-significant decrease for the FGF2 gene and a low significant decrease with PDL2 *p* < 0.05 in comparison to the cisplatin (Figure 5B). On the other side, the activity of PdL1LNP and PdL2LNP revealed a significant decrease in the quantitative gene expression of FGFR2 than cisplatin with *p* < 0.0001 and *p* < 0.01 compared to trastuzumab. Additionally, LNPs-encapsulated Pd(II) complexes (PD_1_LNP and PD_2_LNP) expressed better inhibitory effects for the FGFR2 with *p* < 0.0001 in comparison to cisplatin and *p* < 0.01 in comparison to trastuzumab (Figure 5A). The diminution effect on the FGF2 expression was lower than cisplatin treatment with *p* < 0.01 and *p* < 0.001 for PD1LNP and PD2LNP, respectively, and with *p* < 0.01 for both encapsulated complexes in comparison to trastuzumab (Figure 5B). 

#### 2.3.4. The Enzyme-Linked Immunosorbent Assay (ELISA) for FGFR2, Her2/neu, TGFβ1, and P53

**Figure 6:**
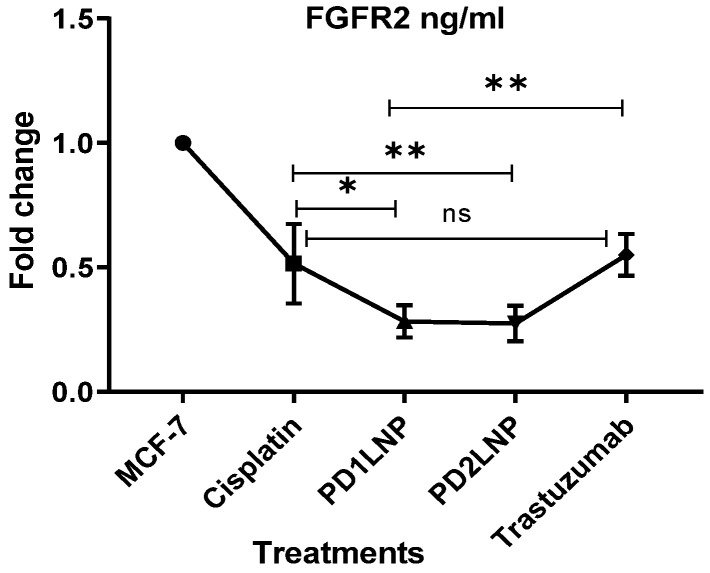
The effect of the LNP-encapsulated Pd(II) complexes in the form of PdL_1,2_LNPs treatment on the FGFR2 protein levels in MCF-7 carcinoma in comparison to cisplatin and trastuzumab. Data are presented as mean ± SE. * *p* < 0.05, ** *p* < 0.01, and ns, non-significance.

The activity of the LNPs-encapsulated Pd(II) complexes on the protein expression of the fibroblast growth factor receptor revealed a significant decrease from the cisplatin and the trastuzumab that may prove their ability to prevent the FGF2/FGFR2 interaction axis. Pd1LNP and Pd2LNP significantly decrease the FGFR2 receptor better than cisplatin with *p* < 0.05 and 0.01, respectively, and from the trastuzumab with *p* < 0.01 (Figure 6).

**Figure 7:**
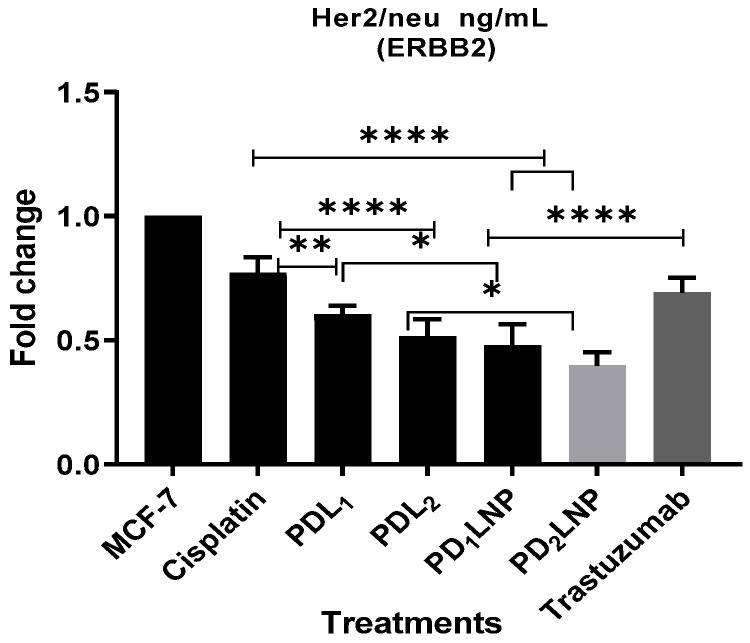
The effect of the LNPs-encapsulated Pd(II) complexes in the form of PdL_1,2_LNPs (or PD_1,2_LNPs) treatments on the Her2/neu protein levels in MCF-7 carcinoma in comparison to cisplatin and trastuzumab. Significance was determined according to mean ± SE. * *p* < 0.05, ** *p* < 0.01, and **** *p* < 0.0001.

On one side, the activity of the PdL_1, 2_ complexes revealed a significant reduction in the Her2/neu with *p* < 0.01 and *p* < 0.0001 compared to the activity of the cisplatin, respectively. Conversely, their LNP encapsulated form exerted more reduction with *p* < 0.05. Moreover, Pd_1,2_LNP revealed a highly significant drop for the Her2/neu quantitative expression in the MCF7 treated cells with a *p*-value < 0.0001 compared to cisplatin and trastuzumab (Figure 7). The extracellular domain of this receptor is where trastuzumab binds to exert its activity, which inhibits HER2 homodimerization and consequently blocks HER2-mediated signaling that confirms their vital role as anticancer Pd(II) drug overcome their produced increased chemotherapy resistance and the widely acknowledged drawbacks of antineoplastic medications based on platinum. 

**Figure 8:**
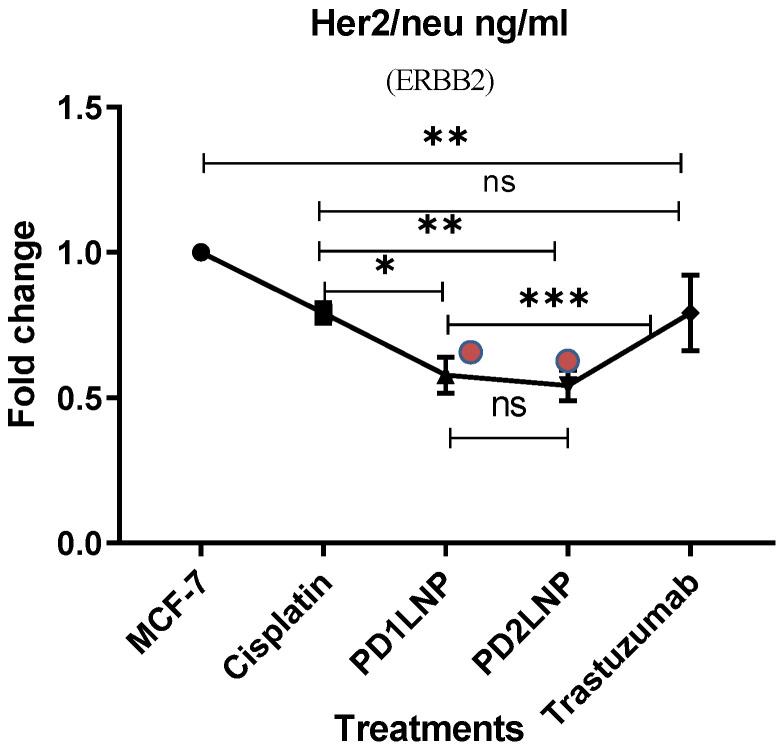
The effect of the lipid-encapsulated Pd(II) complexes in the form of PdL_1,2_LNPs treatment on the TGF-β1 protein levels in MCF-7 carcinoma in comparison to cisplatin and trastuzumab. Significance was according to mean ± SE. * *p* < 0.05, ** *p* < 0.01, *** *p* < 0.001. and ns, non-significance.

The crucial inhibitory roles of the LNP-encapsulated Pd(II) complexes on the transforming growth factorβ in MCF-7 carcinoma were obvious. The activity of the PdL_1,2_LNPs significantly decreased the quantitative expression of TGFβ of MCF-7 carcinoma with *p* < 0.01 from the cisplatin. Moreover, the trastuzumab appeared to have no hormonal activity on the TGFβ1 expression (Figure 8).

**Figure 9:**
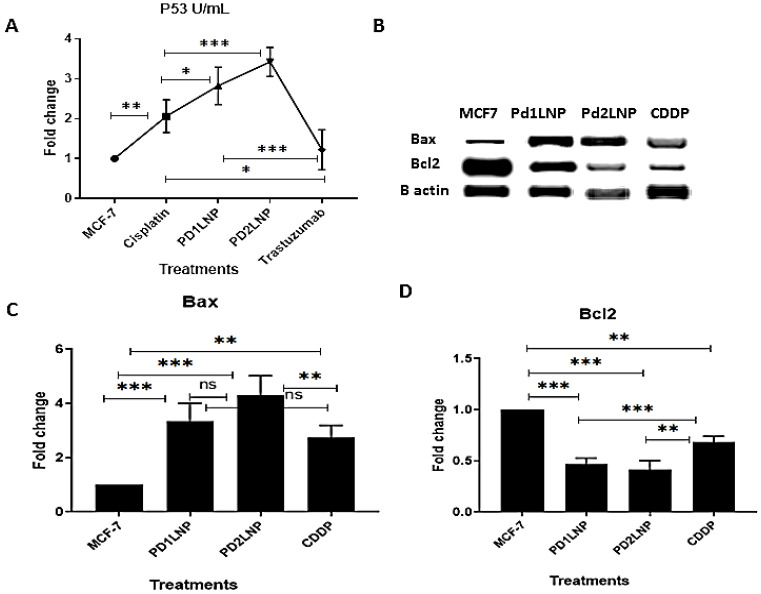
The effect of the lipid encapsulated Pd(II) complexes in the form of PdL_1,2_LNPs treatment on the apoptotic markers in MCF-7 in comparison to cisplatin. (**A**) P53; (**B**) Western blotting of Bax and Bcl2; (**C**,**D**) densitometric analysis of band expression of Bax and Bcl2, respectively. Significacne as mean ± SE. * *p* < 0.05, ** *p* < 0.01, *** *p* < 0.001, and ns, non-significance.

The synthesized encapsulated Pd(II) complexes in PdL1,2LNPs revealed significant upregulation for the levels of the expressed P53 with *p* < 0.05 and *p* < 0.001, respectively, in comparison to cisplatin activity to upregulation with *p* < 0.01 in comparison to the MCF-7 carcinoma cells (Figure 9A). Moreover, to ascertain the activity of the encapsulated Pd (II) complex on the apoptosis mechanisms behavior in MCF-7, Bax and Bcl2 proteins expression was analyzed using Western blotting and revealed their significant changes (Figure 9B). On one side, bax was significantly upregulated after the treatment with *p* < 0.001 for both treatments and CDDP with *p* < 0.01, while there were no significant differences among the synthesized compounds (Figure 9C). Conversely, the synthesized complexes could downregulate the high expression levels of the oncoprotein BCL2 in MCF-7 significantly, with *p* < 0.001 (Figure 9D). 

## 3. Discussion

Breast cancer is a multifactor disease that occurs by abnormal cell signaling pathways due to genetic and epigenetic changes. The development and metastasis of cancer are influenced by several growth factors and their receptors. The accumulation of many genetic alterations is necessary for the gradual transformation of normal cells into extremely malignant descendants. Additionally, somatic mutations are facilitated by germ-line mutations due to DNA repair system failure, leading to the loss of tumor suppressor roles and the induction of oncogene functions [41]. 

Our work’s main objective is to identify safe and easily cellular permeable compound ligands targeting breast cancer (MCF-7) cells using encapsulated Pd(II)L_1,2_LNPs. The activity of the synthesized complexes has been tested for their inhibitory actions for Her2/neu and FGFR2 that can dwindle tumor metastasis and proliferation. The complexes Pd_1,2_LNPs showed their capacity to induce breast cancer cell death mechanisms and proliferation inhibition through different mechanisms.

These results showed that PdL_1_LNP and PdL_2_LNP had a substantial role (85% and 75%, respectively) in triggering significant DNA fragmentation in the MCF-7 cell lines in comparison to cisplatin and trastuzumab with decreased activity at 35% and 10%, respectively. These findings shed light on the relative contribution of Pd complexes to induce selective targets for cancer cell DNA damage and disrupting DNA integrity within MCF-7 cells. Further exploration of the mechanisms underlying these effects could lead to the development of novel therapeutic approaches for cancer treatment.

Moreover, the PdL_1,2_LNPs encapsulated in lipid nanoparticles induced a significant decrease in both FGFR2/FGF2 gene expression and decreased quantitative levels of activity for FGFR2 on the MCF-7. So, the present study was in agreement with the previous study [42], which reported that higher incidence and rapid progression of breast cancer are associated with mutations and deregulated expression in the fibroblast growth factor receptor (FGFR) gene trigger the interaction between luminal invasive ductal cancer (IDC) and its environment to induce disease-independent progression. So, FGFR inhibitors have the potential to overcome therapeutic resistance to estrogen receptor-targeting agents. So, targetting the FGF/FGFR signaling axis inhibition is significantly crucial in drop-off tumor development and progression [43]. Wesche et al. established that smoking, higher caloric intake, and decreased exercise cause carcinogenesis and that the FGF/FGFR axis contributes to breast cancer through FGFR2 signaling dysregulation as a consequence of the accumulation of epigenetic modifications and genetic abnormalities during chronic inflammation [44]. Small-molecule FGFR inhibitors, including PD173074, SU5402, AZD2171, and Ki23057, revealed advantageous properties to prevent cancer proliferation and progression in people with the FGFR2 risk allele through their detrimental activities to interfere with a cytoprotective mechanism against oxidative stress [45]. Additionally, growth factors and chemokines trigger different signaling cascades that interact with one another in the tumor microenvironment, causing cancer to spread [46].

Moreover, overexpressed HER2/neu in breast cancer is related to aggressiveness and a poor prognosis, and its inhibitors have been completely altered by the development of HER2-focused treatments [47]. Moreover, Hanker et al. [48] proposed that the combination of HER2 and FGFR inhibitors be studied prospectively in HER2+ breast cancer patients with somatic alterations of the FGFR pathway. Consequently, inhibitors target both proteins to prove their dual activities and connective actions for the progressive actions of breast cancer cells. So, the effective metal-based anticancer drug activity, the imidazolium-based Pd(II) complexes, may be exerted due to their propensity to target CT-DNA through the formation of adducts that enhance apoptotic mechanisms for cell death [49,50]. The present study revealed that Pd_1,2_ LNP has an intriguing finding regarding the inhibition of Her2/neu quantitative expression in MCF7 cells compared to the activity of cisplatin and trastuzumab. This finding is particularly noteworthy as it highlights their potential action as a promising alternative treatment option that can be used in combination with hormonal chemotherapy to overcome the exerted resistance to trastuzumab and other agents among Her2/neu positive breast cancers. Additionally, the coincidence of inhibitory activities of the synthesized Pd1, 2LNP for the high expression levels of FGFR2 and Her2/neu supported the hypothesis of their switching in the mechanistic pathways and resistance to HER2 inhibitors through its ability to induce phosphorylated HER2 in vivo and in vitro [51,52]. However, Zhao et al. FGFR2 inhibitors added to HER2-positive breast cancer cells after the failure of treatment with the anti-HER2 drug lapatinib suggested a switch in cell addition to signaling inhibitors [53]. Furthermore, the authors showed that FGF5 secreted by cancer-associated fibroblasts (CAF) in the microenvironment might be responsible for the high activation of FGFR2 on the neighboring epithelial cells [54], confirming the potential signaling switch between HER2 and FGFR2 in breast cancer.

Further, the expression of FGFR2 and FGF2 on the gene and quantitative protein levels was compared with the inhibitory effect of PdL_1,2_LNPs on the her2/neu expression, demonstrating their tight relationship to one another and their potential inhibitory actions by the same inhibitor [3]. So, the treatment with synthesized Pd_1,2_LNP as a novel active chemotherapeutic with target treatment for the charged tumor cell has preferred mechanisms with its antitumor activity and is characterized by antiproliferative properties toward different tumor cell lines with actions better than trastuzumab, which only targeted the extracellular domain of the HER-2 protein [36,50]. Siena et al. concluded that the FGF signaling system controls several biological processes, including cell division, migration, and differentiation. The epidermal growth factor receptor family member HER2 controls the proliferation and differentiation of healthy cells and also functions as a tumor-triggering pathway by promoting overexpression [55]. Additionally, it was obvious that the lipid nanoparticles that encapsulated Pd(II) complexes triggered higher activity than the Pd(II) complexes due to their ability to penetrate the cellular membrane and maximize the activity of Pd (II) to form either covalent or non-covalent interactions with the cancer cell DNA either for FGFR2 or HER2/neu genes through the force of electrostatics between positively charged metal complexes and negatively charged DNA [56].

In disagreement with Yasui et al., who concluded that it was impossible to distinguish any variations in FGFR2 or HER2 status based on primary cancer’s location or histological subtype, there were notable variances between the various histological kinds of gastric cancer; however, FGFR2 and HER2 status did not vary according to the main cancer location, due to the small number of FGFR2-positive colorectal cancer patients without full validation of their results [57].

Jovanović et al. confirmed that most Pd(II) complexes have anticancer activity with an antiproliferative role against different types of carcinomas through DNA or protein-binding abilities [50] with obscure mechanisms about their activity as potential novel antineoplastic agents. 

One of the primary and most important functions of the produced chemotherapeutic drugs to be TGF inhibitors was proven through the ability of the encapsulating Pd(II) complexes in PdL_1,2_LNPs to lower TGF-1. Cui et al. demonstrated that targetting TGF-1 expression in suprabasal keratinocytes appears to have multiple effects in mouse skin cancer models [58]. Lowering or inhibition of TGF-1 prevents benign skin tumors from growing and progressing to a highly invasive spindle cell phenotype. These findings imply that TGF-1 actions are biphasic: TGF-1 operates early as a tumor suppressor, likely by preventing the growth of untransformed cells, and later as a tumor promoter by inducing an epithelial-to-mesenchymal transition (EMT). Mani et al. have shown that breast tumor subpopulations with CD44+ cancer stem-like cells (CSC) feature overexpressed TGF-1 and the TGF-I receptor (TGF-R1). Additionally, TGF-R1/2 kinase inhibitors can prevent EMT and trigger the differentiation of CD44+ mammary epithelial cells from mesenchymal to epithelial tissue. Tumor cells, as well as stromal and immune cells linked to the tumor, can produce TGF-ligands [59], and TGFβ-inhibitors have been proposed as antimetastatic therapy among cancer patients.

The present study revealed that the MCF-7 carcinoma cells revealed downregulated expression levels of the most common apoptotic marker, P53. These results hold promise for the development of novel therapeutic strategies that utilize PdL_1,2_LNPs and their encapsulated Pd(II) complexes to specifically target and enhance the expression of P53, potentially leading to more effective cancer treatments in the future. Furthermore, trastuzumab did not significantly increase P53 apoptotic activity, which could be a marker of the drug’s ability to block HER2 homodimerization by binding to an extracellular area of the receptor and preventing HER2-mediated signaling. HER2-expressing cells are thought to be killed via antibody-dependent cellular cytotoxicity [60]. The elevation of the P53 protein to control the cell cycle is one of the primary targeting activities that control the growth of breast cancer. Therefore, TP53 gene mutations are present in around 50% of human breast, colon, lung, liver, prostate, bladder, and skin malignancies [61]. Wildtype P53 prevents cell division when DNA is harmed until the damage is fixed. This prevents the spread of cells with chromatin defects and the emergence of the cancer phenotype. Therefore, TP53 mutations that alter the cell cycle allow cells to lose control over their reproduction, which results in the transmission of faulty DNA to their progeny and the development of cancerous cells [62]. Senescence and cell-cycle arrest are caused by P53 activation when there is enough carcinogenic stress present, which is a fundamental mechanism governing the prevention of carcinogenesis [62]. Oncogenic stimuli usually concentrate on important signaling nodes involved in the regulation of mTOR kinase. P53’s defective expression features in MCF-7 exhibit a variety of cell-toxic signals like genotoxic stress and DNA damage [62]. Additionally, the cell cycle is controlled by the activity of the encapsulating Pd(II) complexes in PdL_1,2_LNPs, which activates the transcription factor P53 as a possible chemotherapeutic agent. The role of the P53 protein in various biological processes, such as cell cycle control, gene expression regulation, aging, and programmed cell death, has been established. These criteria indicate that P53, a critical protein present in many organisms, contributes to the prevention of cancer while protecting the genome [63]. When P53 halts the cell cycle at the G1 and G2 control points in reaction to DNA damage, the DNA-mending proteins can become active. If the damage is irreparable, the Bax gene is triggered, which causes apoptosis [64]. Additionally, the upregulation of Bax proteins by treatment with PD1,2LNP complexes accelerated the intrinsic apoptosis and led to overcoming cancer cells' resistance to various cytotoxic chemotherapy medications [54]. Additionally, the significant downregulation of the Bcl2 proteins was remarkable in magnifying the beneficial role of Pd (II) treatment through their activity to direct cancer cells to apoptotic mechanisms. Bcl2 enhances the keeping of the mitochondrial membrane integrity and prevents apoptotic particle release through Bax/BAK oligomerization inhibition, leading to the deactivation of pro-apoptotic proteins like Bax [65]. 

The activity of palladium(II), a metal that belongs to the same chemical family as platinum, is the cancer chemotherapeutics of choice [66]. Keter et al. reported that complexes comprising platinum(II) and palladium(II) induce apoptosis in cancer cells by enhancing cell death and upregulating the G1 phase [67]. Additionally, the Pd complex induced mitochondrial malfunction that resulted in the loss of mitochondrial membrane potential, the production of reactive oxygen species, and the release of cytosolic cytochrome c, which activated the caspase-9 and caspase-3 proteins and ultimately led to programmed cell death. Additionally, it was noted that certain palladium complexes significantly increase DNA damage and trigger apoptosis [68].

## 4. Materials and Methods

The electronic supplementary materials (ESM†) serve as a repository of detailed information about the chemical substances, reagents, and their suppliers, as well as the instrumentations employed in this study. In addition, the protocols used for the preparation of (*R*,*R*)-1,2-diaminocyclohexane (*R*,*R*-dach), 5-chlormethyl-*o*-vanillin (1), and 3-(vanillyl)-1-butylimidazolium ionic liquids, VAN(*^n^*BuIm^+^-X^–^) (2a,b), were depicted in the ESM†.

### 4.1. Synthesis of R,R-H_2_(VAN)_2_dach(nBuIm^+^-X^–^)_2_ Ligands (H_2_L_1,2_)

A solution of *R*,*R*-dach (2.0 mmol) in 10 mL ethanol was mixed with an ethanolic solution of imidazolium salt Val(*^n^*BuIm^+^-X^–^) (2a,b) (4.0 mmol, 20 mL) into a Schlenk flask under a N_2_ atmosphere and vigorous stirring, and solvent evaporation yielded viscous residue for ligand synthesis (H_2_L_1,2_). This residue was made solid by adding ethyl acetate (AcOEt) and refrigerating overnight. The AcOEt was then discarded, and the crude products were purified with a series of washes with Et_2_O and a mixture of MeOH and Et_2_O to remove any leftover unreacted substances; each wash was performed in triplicate. The leftover material was dissolved in MeOH before being reprecipitated with a slow addition of EtOAc to produce pure ligands, which were then collected via filtration and dried for an entire night in a vacuum at 40 °C. The obtained ligands were physicochemically characterized as follows;

N,N′-Bis[5-((1-butylimidazolium)methyl)-o-vanillylidene)]-cyclohexanediamine dichloride (H_2_L_1_): Obtained as a yellow-orange solid with a yield of 89.7% and mp 71–72 °C. FTIR (KBr, cm^−1^): 3437 (s, br, *ν*_(Ph-OH)_), 3075 (s, sh), 2934 (m, sh) 1635 (vs, sh, *ν*_(azomethine)_), 1558, 1461, 1377 (m, sh), 1271 (s, sh), 1163 (s, sh), 752 (m, sh), 620 (w, sh). ^1^H NMR (200 MHz, DMSO-*d*_6_) δ 9.20 (s, 2H, 2 OH), 8.52 (s, 2H, 2 HC=N), 7.81 (d, *J* = 1.6 Hz, 1H, Im-H), 7.79 (d, *J* = 1.5 Hz, 4H, 4 Im-H), 7.22 (dd, *J* = 14.0, 2.0 Hz, 1H, Im-H), 7.14 (d, *J* = 1.9 Hz, 2H, 2 Ar-H), 7.08 (d, *J* = 1.9 Hz, 2H, 2 Ar-H), 5.28 (s, 4H, Ar-CH_2_-), 4.16 (t, *J* = 7.1 Hz, 4H, 2 N-CH_2_-CH_2_-), 3.77 (s, 6H, OCH_3_), 3.55–3.49 (m, 2H, 2 Hex-H), 1.85–1.70 (m, 8H, 8 Hex-H), 1.66–1.45 (m, 4H, 2 N-CH_2_-CH_2_-), 1.35–1.18 (m, 4H 2 N-CH_2_-CH_2_-CH_2_-), 0.90 (t, *J* = 7.3 Hz, 6H, 2 N-CH_2_-CH_2_-CH_2_-CH_3_). ^13^C NMR (126 MHz, DMSO-*d*_6_) δ (ppm): 164.90 (C=N), 161.33 (C-OCH_3_), 136.22 (C-OH), 133.14 (Im-C), 132.42 (Ar-C), 125.02 (Ar-C), 123.07 (Im-C), 122.70 (Im-C), 118.74(Ar-C), 117.63(Ar-C), 71.56 (Hex-C), 65.29 (Ar-CH_2_-), 51.70 (C-OCH_3_), 49.02 (N-CH_2_-CH_2_-), 36.13 (N-CH_2_-CH_2_-), 31.62 (Hex-C), 31.04 (Hex-C), 19.17 (N-CH_2_-CH_2_-CH_2_-), 13.61 (N-CH_2_-CH_2_-CH_2_-CH_3_). +ve mode ESI-MS: *m*/*z* 687.3 and 328.2 ([C_38_H_52_ClN_6_O_4_]^+^ and [C_38_H_52_N_6_O_4_]^2+^, [M–Cl^–^]^+^ and [M–2Cl^–^]^2+^, respectively). Anal. Calcd. for C_38_H_52_Cl_2_N_6_O_4_ (M = 727.77): C, 62.71; H, 7.20; N, 11.55; Found: C, 62.63; H, 7.29; N, 11.38.

N,N′-Bis[5-((1-butylimidazolium)methyl)-o-vanillylidene)]-cyclohexanediamine bis-(tetrafluoro-borate) (H_2_L_2_): Obtained as a yellow solid with a yield of 88.3% and mp 82–83 °C. FTIR (KBr, cm^−1^): 3439 (m, br, *ν*_(Ph-OH)_), 3112 (s, sh), 2937 (m, sh) 1634 (vs, sh, *ν*_(azomethine)_), 1564, 1468, 1353 (m, sh), 1276 (s, sh), 1168 (s, sh), 1059 (vs, sh, *ν*_(BF4)_), 753 (m, sh), 622 (w, sh). ^1^H NMR (200 MHz, DMSO-*d*_6_) δ 9.18 (s, 2H, 2 OH), 8.50 (s, 2H, 2 HC=N), 7.81 (d, J = 1.6 Hz, 1H, Im-H), 7.77 (d, J = 1.5 Hz, 4H, 4 Im-H), 7.21 (dd, J = 14.8, 2.0 Hz, 1H, Im-H), 7.12 (d, J = 1.9 Hz, 2H, 2 Ar-H), 7.06 (d, J = 1.9 Hz, 2H, 2 Ar-H), 5.26 (s, 4H, Ar-CH_2_-), 4.15 (t, J = 7.1 Hz, 4H, 2 N-CH_2_-CH_2_-), 3.75 (s, 6H, OCH_3_), 3.55–3.50 (m, 2H, 2 Hex-H), 1.89–1.73 (m, 8H, 8 Hex-H), 1.64–1.44 (m, 4H, 2 N-CH_2_-CH_2_-), 1.28–1.21 (m, 4H 2 N-CH_2_-CH_2_-CH_2_-), 0.89 (t, J = 7.3 Hz, 6H, 2 N-CH_2_-CH_2_-CH_2_-CH_3_). ^13^C NMR (126 MHz, DMSO) δ (ppm): ^13^C NMR (126 MHz, DMSO-d_6_) 165.15 (C=N), 152.92 (C-OCH_3_), 148.87 (C-OH), 136.16 (Im-C), 123.99 (Ar-C), 123.84 (Ar-C), 123.00 (Im-C), 122.72 (Im-C), 117.96 (Ar-C), 115.16 (Ar-C), 70.89 (Hex-C), 56.16 (Ar-CH_2_-), 52.17 (C-OCH_3_), 49.02 (N-CH_2_-CH_2_-), 32.95 (N-CH_2_-CH_2_-), 31.61 (Hex-C), 31.04 (Hex-C), 19.16 (N-CH_2_-CH_2_-CH_2_-), 13.61 (N-CH_2_-CH_2_-CH_2_-CH_3_). ^19^F NMR (471 MHz, DMSO-d_6_) δ (ppm): −148.67 (singlet). ^11^B NMR (96 MHz, DMSO-d_6_) δ (ppm): δ −1.30 (singlet). +ve mode ESI-MS: m/z 743.6 and 328.2 ([C_38_H_52_BF_4_N_6_O_4_]^+^ and [C_38_H_52_N_6_O_4_]^2+^, [M–BF_4_^–^]^+^ and [M–2BF_4_^–^]^2+^, respectively). Anal. Calcd. for C_38_H_52_B_2_F_8_N_6_O_4_ (M = 830.48): C, 54.96; H, 6.31; N, 10.12; Found: C, 54.85; H, 6.29; N, 10.08.

### 4.2. Synthesis of Pd(II)-(VAN)_2_dach Complexes (PdL_1,2_)

Initially, the PdCl_2_ solution was prepared by dissolving 0.126 g of PdCl_2_ (1 mmol) in 5 mL of EtOH containing 1 mL of conc HCl. After that, this solution was added gradually to an ethanolic solution of ligand (1 mmol, 10 mL). The reaction blend was then heated under reflux while stirring for 5 h. Following the vacuum-assisted solvent evaporation, an oily residue was left behind that was then solidified by adding petroleum ether (40–60) and refrigerating overnight. The obtained Pd(II)-(VAN)_2_dach complexes (**4a,b**) were collected via filtration and purified by washing three times with an ice-cold MeOH-Et2O (1:3) mixture. The obtained ligands were physicochemically characterized as follows:

Pd(VAN)_2_dach(^n^BuIm^+^-Cl^–^)_2_ complex (PdL_1_): Obtained as a brown solid with a yield of 69.2%. FTIR (KBr, cm^−1^): 3082 (s, sh), 2934 (m, sh) 1618 (s, sh, *ν*_(azomethine)_), 1556, 1460, 1347 (m, sh), 1264 (s, sh), 1172 (m, sh), 752 (m, sh), 642 (w, sh), 619 (w, sh), 556 (w, sh), 542 (w, sh), 498 (w, sh), 463 (w, sh), 434 (w, sh). +ve mode ESI-MS: *m*/*z* 796.6 and 385.5 ([C_38_H_50_ClN_6_O_4_Pd]^+^ and [C_38_H_50_CN_6_O_4_Pd]^2+^, [M–Cl^–^]^+^ and [M–2Cl^–^]^2+^, respectively). Anal. Calcd. for C_38_H_50_Cl_2_N_6_O_4_Pd (M = 832.18): C, 54.85; H, 6.06; N, 10.10; Found: C, 54.76; H, 6.13; N, 9.98.

Pd(VAN)_2_dach(^n^BuIm^+^-BF_4_^–^)_2_ complex (PdL_2_): Obtained as a reddish-brown solid with a yield of 64.9%. FTIR (KBr, cm^−1^): 3111 (s, sh), 2937 (m, sh) 1616 (s, sh, *ν*_(azomethine)_), 1559, 1460, 1350 (m, sh), 1268 (s, sh), 1157 (s, sh), 1059 (vs, sh, *ν*_(BF4)_), 760 (m, sh), 643 (w, sh), 617 (w, sh), 571 (w, sh), 542 (w, sh), 498 (w, sh), 466 (w, sh), 433 (w, sh). +ve mode ESI-MS: *m*/*z* 848.0 and 385.5 ([C_38_H_50_BF_4_N_6_O_4_Pd]^+^ and [C_38_H_50_CN_6_O_4_Pd]^2+^, [M–BF_4_^–^]^+^ and [M–2BF_4_^–^]^2+^, respectively). Anal. Calcd. for C_38_H_50_B_2_F_8_N_6_O_4_Pd (M = 934.88): C, 48.82; H, 5.39; N, 8.99; Found: C, 48.80; H, 5.42; N, 8.88.

### 4.3. Preparation of PdL_1,2_ Complexes-Loaded LNPs (PdL_1,2_LNPs)

The previously reported microemulsion process [36], with a few minor alterations, was used to prepare LNPs employing stearyl alcohol (SA) as an internal phase, soya lecithin (SL) as the surfactant, cetyltrimethylammonium bromide (CTAB) as a co-surfactant, and milli-Q water (MQW) as the continuous phase. Initially, a pre-heated SA (10 mg, 20% *w*/*w*) at 90.0 °C was used to thoroughly solubilize a mixture of SL (5 mg, 10% *w*/*w*) and PdL_1,2_ (0.4 mg, 0.8% *w*/*v*) under vigorous stirring. The aqueous phase (33.5 mL) of the co-surfactant CTAB (0.86 mg, 2.5% *w*/*v*) was then heated to 80 °C before being incorporated into the melted lipid dispersion. To prepare the indented microemulsion, the content was first homogenized for 5 min at 25,000 rpm using an UltraTurrax^®^ T10 homogenizer (IKA, Königswinter, Germany) and then ultrasonically treated for 90 s at 40% power and 60% pulse frequency using a probe sonicator. To separate PdL_1,2_LNPs, the obtained microemulsion was disseminated into MQW at 2–5 °C for 10 min while being mechanically stirred (12,000 rpm).

### 4.4. Characterization of PdL_1,2_LNPs

Dynamic light scattering (DLS, Zetasizer Nano NS, Malvern Instruments) was used to measure the mean hydrodynamic diameter (MHD, nm) and polydispersity index (PDI). On the other hand, the zeta potential (ZP) is an important parameter that characterizes the surface charge of PdL_1,2_LNPs in a solution. It provides valuable information about the stability and behavior of colloidal systems. In this context, the ZP of the complexes was measured using an electrophoretic mobility technique utilizing the Zetasizer Nano NS (Malvern Equipment, Malvern, UK). Samples with 1:100 dilutions of each PdL_1,2_LNP dispersion in MQW were used for all measurements. Additionally, the MHDs and ZPs of the PdL_1,2_LNPs in aqueous solutions were measured after 0, 1, 15, 30, and 45 days of storage to validate their physicochemical stability. To ensure the reliability of the findings, data were obtained and represented as mean ± standard deviation.

PdL_1,2_LNPs were analyzed for their morphology using transmission electron microscopy (TEM; H-7650; Hitachi, Japan). All samples were made by diluting them with MQW until they reached the desired concentration. The 100 kV acceleration voltage was used to take the TEM nanographs.

The entrapment efficiency (EE) and loading capacity (LC) on the LNPs were determined by the difference between the complex’s initial concentration and the concentration of the free complex in the dispersion medium. In brief, AMICON ultracentrifuge tubes equipped with an ultrafilter (MWCO-50000) (Millipore, Bedford, MA, USA) were filled with 1 mL of PdL_1,2_LNPs dispersion (200 μg/mL). The tubes were spun for 15 min at room temperature at 3200× *g* in an Eppendorf centrifuge (5810R, Hamburg, German). The filtrate was gathered and analyzed for the concentration of free complexes using the UV spectrophotometric method at λ_max_ 490 nm. To determine the concentration of the free complex in the aqueous solution, a calibration curve was constructed using standard aqueous solutions of each complex. The equation employed to fit the curve within a specific range of complexes was used to estimate the free complex present in the aqueous solution. Following are the equations (Equations (1) and (2)) used to determine the EE and LC:

(1) $$EE\%=\frac{amount\;of\;initial\;complex-the\;amount\;of\;free\;complex}{\;amount\;of\;initial\;complex}\times 100\;$$

(2) $$LC\%=\frac{amount\;of\;initial\;complex-amount\;of\;free\;complex}{\;weight\;of\;{PdL}_{1,2}LNPs}\times 100$$

### 4.5. In Vitro PdL_1,2_ Release from PdL_1,2_LNPs

The dialysis technique was employed to investigate the in vitro PdL_1,2_ release kinetics [15]. In brief, a 5 mL solution of PdL1,2-loaded LNPs at a concentration of 200 μg/mL was introduced into a dialysis tube (Spectra/por^®^ dialysis tubes, MWCO 25,000). The tube was sealed at both ends and inspected for potential leaks. It was then immersed in 100 mL of phosphate-buffered saline (PBS) solutions with pH values of 7.4 and 5.0. The media with the dialysis tube containing PdL_1,2_LNPs was agitated in a dark environment using a magnetic stirrer (100 rpm). Samples (2 mL) of the released medium were withdrawn at specific time intervals (0.5, 1.5, 3, 6, 12, 24, and 48 h) and subjected to analysis using UV–Vis spectroscopy at 490 nm. The previously extracted samples were substituted with 2 mL of a fresh PBS solution to maintain a consistent volume of the releasing solution. The complex release profiles were repeated three times, and Equation (3) [15] was used to determine the percentage of cumulative release (CR) over time.

(3) $$CR\%=\frac{Initial\;amount\;of\;complex-the\;released\;amount\;of\;complex}{\;initial\;amount\;of\;complex}\times 100$$

### 4.6. In Vitro Cytotoxicity Studies

#### 4.6.1. Cell Cultures

Human tumor cell lines MCF-7 (breast cancer) and immortal Hela cells were provided by the VACSERA Tissue Culture Unit and obtained and grown on RPMI-1640 media with 10% FBS, 1% L-glutamine, a HEPES buffer, and the addition of 50 g/mL gentamycin. Cells were kept at 37 °C and 5% CO_2_. Cell viability and cell morphology were both used to assess toxicity. The control cells were given DMSO at a concentration of 0.5% to determine toxicity.

#### 4.6.2. Cytotoxic Effect Assay and Cell Proliferation using MTT

The cytotoxic effects of PdL_1,2_LNPs were evaluated using an in vitro cytotoxicity assay, following guidelines from the Regional Center for Mycology and Biotechnology. Cell growth was observed using MTT, and cell processing was performed using different DDP concentrations. After 48 h, 10 μL of the MTT solution was injected into each pore, and absorbance was measured using a microplate reader. Three groups used a repeated experiment approach for each medication. The repeated experiment of each drug was adopted for three groups. Linear regression was adopted to calculate half maximal inhibitory concentration (IC_50_).

$$Cell\;death\;rate\;\left(\%\right)=\left(1-\frac{{OD}_{sample}}{{OD}_{control}}\right)\times 100\%$$

where, at a test wavelength of 570 nm and a reference wavelength of 655 nm, the optical density (OD) values for the sample and control have been measured.

#### 4.6.3. Cell Viability Assay

At a density of 2000 cells per well, MCF-7 cells were planted in 96-well plates with a black, transparent bottom. The media was changed the following day to 100L of media with 1% charcoal-stripped serum (CSS). Cell nuclei were stained at 37 °C for 20 min after 6 days with 10 g/mL Hoechst 33342 (Thermo Fisher Scientific, Waltham, MA, USA). The (IX70; Olympus, Tokyo, Japan) was used to count fluorescent nuclei.

### 4.7. Comet Assay for DNA Damage Detection

The protocol described by Singh et al. [69] was slightly modified by Blasiak et al. [70] and was used for the comet assay. The slides were examined at a magnification of 40× using an inverted fluorescence microscope (IX70; Olympus, Tokyo, Japan) equipped with a 549 nm excitation filter and a 590 nm barrier filter. With a brilliantly fluorescent head and a lengthy, brilliantly fluorescent tail that were separated from one another during electrophoresis, damaged cells resembled comets. Images were analyzed using Image J (IJ 1.46r) to count damaged cells.

### 4.8. The Enzyme-Linked Immunosorbent Assay (ELISA) for Her2/neu, FGFR2, TGFβ1, and P53

The cell medium was collected and centrifuged to eliminate cellular debris after CDDP, PdL_1,2_LNPs, and trastuzumab were administered to cells in 6-well plates for three days while they were in the growth medium. Cell numbers for each treatment group were calculated. Her2/neu (Life Technologies Corporation, Invitrogen, Carlsbad, CA, USA, Cat#EHERBB2), FGFR2 (Cat# 12828 cell signaling technology, USA), TGFβ1 (Thermo Fisher USA, Cat#PHG9214), and P53 (Novus Biologicals, Centennial, CO, USA, NB200-103). An ELISA was carried out following the manufacturer’s recommendations. Readings were converted to cell numbers, and the fold change versus untreated MCF-7 in the control group was calculated.

### 4.9. Western Blotting Analysis of Bax and Bcle Proteins in MCF-7 Cells

The cell lysates were washed with PBS and lysed using the SDS-PAGE loading buffer, which contained 2 g/L SDS and 50 mmol/L Tris-HCl pH 6.8 and 100 mmol/L dithiothreitol. The sample application was subjected to SDS-PAGE analysis. Bax antibody (Santa Cruz Biotechnology, Inc., Dallas, TX, USA, CAT#sc-7480) and Bcl2 (Santa Cruz Biotechnology, Inc. USA CAT# sc-7382). The appropriate horse radish-peroxidase (HRP) conjugated anti-rabbit IgG (Fc) was then incubated on the membranes after they had been blocked with 50 mL/L skim milk and 1 g/L Tween 20, probed with anti-Bax polyclonal rabbit antibody following the manufacturer’s instructions, and washed with PBS and 2 g/L Tween 20. Following washing, the membranes were developed using DAB reagents according to the instructions provided by the manufacturer (Dako Co., Santa Clara, CA, USA). To ensure uniform protein loading, the amount of β-actin was utilized as a control.

### 4.10. Quantitative Real-Time (qPCR) Estimation FGFR2 and FGF2

Reverse transcriptase was utilized in the study to create cDNA for quantitative real-time PCR. An SV total RNA isolation method was used to collect total RNA from MCF-7 treated cells. Version 3.1 of the Applied Biosystems software was used to perform real-time qPCR amplification with SYBR Green I. All cDNA samples were amplified in duplicate using the qPCR assay, including the ATG gene expression, internal control (GAPDH gene expression), and non-template control (water). The master mix in the PCR tubes was well mixed without bubbling. The metallothionein and housekeeping genes were examined, and the Ct values for each gene were recorded on the PCR data sheet. Gene expression evaluation in a subpar control sample and a correlation between the target genes and the internal control’s expression were discovered. The RQ was calculated according to the following equation: Δ Ct = Ct _assessed gene_ − Ct _reference gene_. Δ Δ Ct = Δ Ct _sample_ − Ct _internal control gene_. RQ = 2^−(Δ Δ Ct)^. primers used for FGFR2: F-5′-CACGGACAAAGAGATTGAGGTTCT-3′, R-5′-CCGCCAAGCACGTATATTCC-3; FGF2: exon 10 F-5′AACAACACGCCTCTCTTCAACG3′, R-5′GTTGCTTTGGGCAAGTGGTC3′; GADPH: F-5′-ACGGGAAGCTCACTGGCA TGG-3′, R-5′-GGTCCACCACCCTGTTGCTGTA-3′.

### 4.11. Statistical Analyses

Experimental measurements were analyzed in quadruplicate. Statistical comparisons between different experimental groups were performed using a one-way analysis of variance (ANOVA) test, followed by Tukey’s multiple comparison post hoc test for pairwise comparisons. Prism 7 software (GraphPad, San Diego, CA, USA) was used to calculate the significance of differences between experimental groups, with a *p <* 0.05 significance criterion. Protein expression densitometry analysis using Western blotting was quantified using the Image J software (ImageJ—win64 2.0). The data were shown as the mean ± standard deviation (SD).

## 5. Conclusions

Two new bis-(imidazolium–vanillylidene)-(*R*,*R*)-diaminocyclohexane ligands (H_2_(VAN)_2_dach, H_2_L_1,2_) and their Pd(II) complexes (PdL_1_ and PdL_2_) were successfully synthesized and structurally characterized. Subsequently, these complexes were encapsulated by lipid nanoparticles (LNPs) to produce (PdL_1_LNP and PdL_2_LNP) that are physicochemically and morphologically characterized with the goal of developing novel effective and safe anti-breast cancer chemotherapeutic drugs. LNPs are considered “Nano safe” carriers due to their great bioavailability while delivering the targeted chemotherapeutic Pd (II) molecules to stop the growth of the tumor because they are built from physiologic and/or biodegradable lipids. These effects came about as a result of the ability to reduce her2/neu expression and maintain the balance of the FGFR2/FGF2 axis. Additionally, the upregulated P53 is considered evidence of directing cancer to programmed apoptotic mechanisms through the activity of Pd (II) to break DNA strands and inhibit the proliferative characteristics of cancer cells. Additionally, Pd_1,2_LNP complexes boosted the production of the Bax protein, which increased the apoptosis rate and helped cancer cells overcome drug resistance. The benefits of Pd (II) treatment were magnified by their ability to down-regulate the expression of Bcl2 proteins, which guided cancer cells toward apoptotic pathways. Bcl2 expression prevents BAX/BAK oligomerization and enhances the integrity of the mitochondrial membrane, preventing the apoptotic mechanisms. So, innovatively manufactured Pd(II) encapsulated with lipid nanoparticles may open up a new era for chemotherapeutics with low toxicity and biocompatibility for cancer plasma membranes. Based on the promising findings presented in the present work, our future work will use imidazolium-supported Pd(II) _1,2_ *o*-vanillylidene/LNPs complexes on different tumor cell lines and investigate their role on mitochondrial dynamic proteins activity and tumor progression cell cycle factors parallel with the inhibitory mechanisms for her2/neu and FGFR subunits.

## Figures and Tables

**Table 1:** Values of IC50 ± SD (μg/mL) and selectivity index (SI) of the new materials against MCF-7 and HSF cell lines.

Treatment	Cell Line	PdL_1_	PdL_2_	PdL_1_LNPs	PdL_2_LNPs	CDDP	TRZ
IC_50_ (μg/mL)	MCF-7	16.28 ± 0.56	23.72 ± 1.25	1.93 ± 0.29	2.03 ± 0.21	5.51 ± 0.31	2.41 ± 0.27
	Hela	53.85 ± 0.78	72.34 ± 0.89	38.96 ± 1.73	41.28 ± 1.53	75.25 ± 3.75	19.72 ± 1.89
SI (%) *		3.31	3.05	20.19	20.33	13.66	8.18

* SI (%) = IC_50_ against healthy cell/IC_50_ cancer healthy cell.

## Data Availability

Data is contained within the article and supplementary material.

## Supplementary Materials

The following supporting information can be downloaded at https://www.mdpi.com/article/10.3390/ph16121711/s1, 1. Materials and instrumentations; 2. Synthesis 2.1. Synthesis of *R,R-*1,2-diaminocyclohexane tartrate salt; 2.2. Synthesis of *R,R-*1,2-diaminocyclohexane; 2.3. Synthesis of 3-methoxy-5-chloromethyl-2-hydroxybenzaldehyde (1); 2.4. Synthesis of 3-(3-(methoxy)-5-formyl-4-hydroxybenzyl)-1-nbutylylimidazolium chloride (2a); 2.5. *Anion metathesis (2b)*. Figure S1: ^1^HNMR spectrum of imidazolium–vanillylidene ligand (H_2_L_1_) (200 MHz, DMSO-*d*_6_); Figure S2: ^1^HNMR spectrum of imidazolium–vanillylidene ligand (H_2_L_2_) (200 MHz, DMSO-*d*_6_); Figure S3: ^13^C NMR spectrum of imidazolium–vanillylidene ligand (H_2_L_1_) (125 MHz, CDCl_3_); Figure S4: ^13^C NMR spectrum of imidazolium–vanillylidene ligand (H_2_L_2_) (125 MHz, DMSO-*d*_6_); Figure S5: ^11^B NMR spectrum of imidazolium–vanillylidene ligand (H_2_L_2_) (96 MHz, DMSO-*d*_6_); Figure S6: ^19^F NMR spectrum of imidazolium–vanillylidene ligand (H_2_L_2_) (470 MHz, DMSO-*d*_6_); Figure S7: FTIR spectrum of imidazolium–vanillylidene ligand (H_2_L_1_); Figure S8: FTIR spectrum of imidazolium–vanillylidene ligand (H_2_L_2_); Figure S9: FTIR spectrum of Pd(II) imidazolium–vanillylidene complex (PdL_1_); Figure S10: FTIR spectrum of Pd(II) imidazolium–vanillylidene complex (PdL_2_).
