# Correction: Awaji et al. Chemotherapeutic Activity of Imidazolium-Supported Pd(II) *o*-Vanillylidene Diaminocyclohexane Complexes Immobilized in Nanolipid as Inhibitors for HER2/neu and FGFR2/FGF2 Axis Overexpression in Breast Cancer Cells. *Pharmaceuticals* 2023, *16*, 1711

**DOI:** 10.3390/ph18091287

**Published:** 2025-08-28

**Authors:** Aeshah A. Awaji, Moustafa A. Rizk, Raiedhah A. Alsaiari, Norah F. Alqahtani, Fatima A. Al-Qadri, Ali S. Alkorbi, Hani S. Hafez, Reda F. M. Elshaarawy

**Affiliations:** 1 Department of Biology, Faculty of Science, University College in Taymaa, University of Tabuk, Tabuk 71491, Saudi Arabia; 2 Department of Chemistry, Faculty of Science and Arts at Sharurah, Najran University, Sharurah 68342, Saudi Arabia or moustafarizk@science.suez.edu.eg (M.A.R.); raalsayari@nu.edu.sa (R.A.A.); fatimaalqadri@gmail.com (F.A.A.-Q.); assalem@nu.edu.sa (A.S.A.); 3 Department of Chemistry, College of Science, University of Jeddah, Jeddah 21589, Saudi Arabia; nfalqahtani@uj.edu.sa; 4 Zoology Department, Faculty of Science, Suez University, Suez 43533, Egypt; 5 Department of Chemistry, Faculty of Science, Suez University, Suez 43533, Egypt; 6 Institut für Anorganische Chemie und Strukturchemie, Heinrich-Heine Universität Düsseldorf, 40204 Düsseldorf, Germany

## Error in Figure

In the original publication [1], there was a mistake in Figure 4 as published. This was an error related to image production during the authors’ preparation of the figures. The corrected Figure 4 appears below. The authors state that the scientific conclusions are unaffected. This correction was approved by the Academic Editor. The original publication has also been updated.

## Figures and Tables

**Figure 4:**
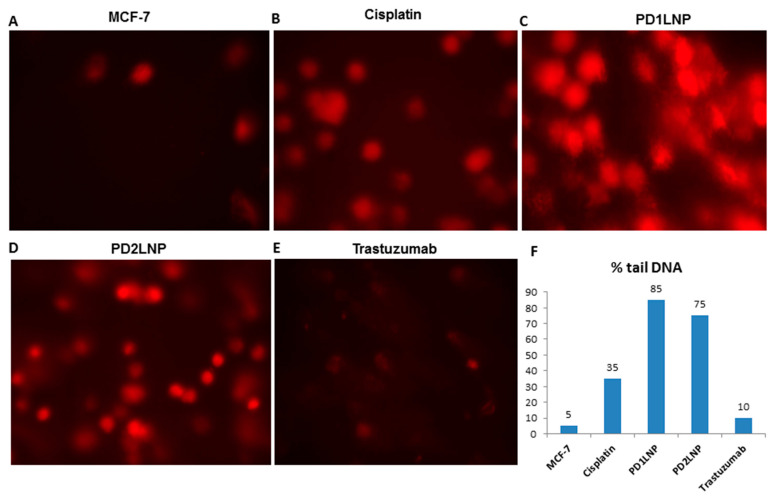
(**A**–**F**) Quantitative measure for 100 cells on a scale from 0 to 400.
