# Supplementary material for: Chemotherapeutic Activity of Imidazolium-Supported Pd(II) o-Vanillylidene Diaminocyclohexane Complexes Immobilized in Nanolipid as Inhibitors for HER2/neu and FGFR2/FGF2 Axis Overexpression in Breast Cancer Cells
Source: Pharmaceuticals (Basel). 2023 Dec 11;16(12):1711. doi: 10.3390/ph16121711 (PMC10747766; doi:10.3390/ph16121711)
Supplement: Supplementary file 1 [file pharmaceuticals-16-01711-s001.zip › pharmaceuticals-2697821-supplementary-done.pdf]

Chemotherapeutic Activity of Imidazolium-Supported Pd(II) *o*-Vanillylidene Diaminocyclohexane Complexes Immobilized in Nanolipid as Inhibitors for HER2/neu and FGFR2/FGF2 Axis Overexpression in Breast Cancer Cells

Aeshah A. Awaji [1], Moustafa A. Rizk [2], Raiedhah A. Alsaiari [2], Norah F. Alqahtani [3], Fatima A. Al-Qadri [2], Ali S. Alkorbi [2], Hani S. Hafez [4,*] and Reda F. M. Elshaarawy [5,6,*]

[1] Department of Biology, Faculty of Science, University College in Taymaa, University of Tabuk, Tabuk 71491, Saudi Arabia; aawaji@ut.edu.sa

[2] Department of Chemistry, Faculty of Science and Arts at Sharurah, Najran University, Sharurah 68342, Saudi Arabia; marizk@nu.edu.sa or moustafarizk@science.suez.edu.eg (M.A.R.); raalsayari@nu.edu.sa (R.A.A.); fatimaalqadri@gmail.com (F.A.A.-Q.); assalem@nu.edu.sa (A.S.A.)

[3] Department of Chemistry, College of Science, University of Jeddah, Jeddah 21589, Saudi Arabia; nfalqahtani@uj.edu.sa

[4] Zoology Department, Faculty of Science, Suez University, Suez 43533, Egypt

[5] Department of Chemistry, Faculty of Science, Suez University, Suez 43533, Egypt

[6] Institut für Anorganische Chemie und Strukturchemie, Heinrich-Heine Universität Düsseldorf, 40204 Düsseldorf, Germany

* Correspondence: hani.hafez@suezuniv.edu.eg (H.S.H.); reda.elshaarawy@suezuniv.edu.eg (R.F.M.E.)

Contents:

1- **Materials and instrumentation**

2- **Synthesis and characterization of the key starting materials**

3- **Figures**

1. Materials and instrumentation

Chemicals were obtained from the following suppliers and used without further purification: salicylaldehyde, 2-ethylphenol, (±)-trans-1,2-diaminocyclohexane and anhydrous $MgCl_2$ (Sigma–Aldrich), paraformaldehyde (Roth), 1-butylimidazole (Alfa Aesar), triethylamine (Et_3N) and anhydrous $ZnCl_2$ (Grüssing GmbH) and palladium(II) chloride $PdCl_2$ (Acros).

Reagents In vitro anticancer activity: Dimethylsulphoxide (DMSO), crystal violet and Trypan blue dye were purchased from Sigma Chemical Co. (St. Louis, Mo, USA). Dulbecco's modified Eagle's medium (DMEM), Roswell Park Memorial Institute medium (RPMI-1640), Fetal Bovine Serum (FBS), 4-(2-Hydroxyethyl)piperazine-1-ethanesulfonic acid (HEPES) buffer solution, L-glutamine, gentamycin and 0.25% Trypsin- EDTA were obtained from Lonza.

Melting points were measured using a BÜCHI Melting point B-540 apparatus; all melting points were measured in open glass capillaries and are uncorrected. Elemental analyses for C, H, N, were performed with a Perkin–Elmer 263 elemental analyzer. FT-IR spectra were recorded on a BRUKER Tensor-37 FT-IR spectrophotometer in the range 400–4000 cm^{-1} as KBr disc in the 4000-550 cm^{-1} region with 2 cm^{-1} resolution or with an ATR (attenuated total reflection) unit (Platinum ATR-QL, diamond). For signal intensities the following abbreviations were used: br (broad), sh (sharp), w (weak), m (medium), s (strong), vs (very strong). UV/Vis spectra were measured at 25 °C in ethanol (10^{-5} mol/L) on a Shimadzu UV-2450 spectrophotometer using quartz cuvettes (1 cm). NMR-spectra were obtained with a Bruker Avance DRX200 (200 MHz for ^{1}H) or Bruker Avance DRX500 (125, 202 and 470 MHz for ^{13}C, ^{31}P and ^{19}F respectively) spectrometer with calibration to the residual proton solvent signal in DMSO-d_6 (^{1}H NMR: 2.52 ppm, ^{13}C NMR: 39.5 ppm), $CDCl_3$ (^{1}H NMR: 7.26 ppm, ^{13}C NMR: 77.16 ppm) against TMS ($\delta = 0.00$ ppm) for ^{1}H and ^{13}C, 85% phosphoric acid ($\delta = 0.00$ ppm) for ^{31}P and $CFCl_3$ ($\delta = 0.00$ ppm) for ^{19}F NMR. Multiplicities of the signals were specified s (singlet), d (doublet), t (triplet), q (quartet) or m (multiplet). The ESI-MS of the synthesized compounds were acquired in the linear mode for positive ions on a UHR-QTOF maXis 4G (Bruker Daltonics) and BRUKER Ultraflex MALDI-TOF instrument equipped with a 337 nm nitrogen laser pulsing at a repetition rate of 10 Hz. The 2+ charge assignment of ions in HR-ESI-MS was confirmed by the m/z = 0.5 difference between the isotope peaks (x, x+1, x+2). Peaks with chlorine showed the isotope ratio $^{35/37}Cl$ = 75.8:24.2.

2. Synthesis and characterization of the key starting materials

2.1. R,R-1,2-diaminocyclohexane tartrate salt

2.2. R,R-1,2-diaminocyclohexane

*2.3. 3-methoxy-5-chloromethyl-2-hydroxybenzaldehyde (**1**).*

*2.4. 3-(3-(methoxy)-5-formyl-4-hydroxybenzyl)-1-nbutylylimidazolium chloride (**2a**).*

*2.5. Anion metathesis (**2b**)*

2.1. R,R-1,2-diaminocyclohexane tartrate salt

L-(+)-tartaric acid (15 g, 100.0 mmol) was dissolved in distilled water (40 ml) and a *rac-trans*-1,2-diaminocyclohexane (24 ml, 200.0 mmol) was added so that the reaction temperature reached 70 °C. To this glacial acetic acid was added (10 ml, 180 mol) so that the reaction temperature reached 90 °C. The resulting slurry was stirred for a further 2 h, and then cooled to 5 °C for 2 h. The resulting precipitate was collected by vacuum filtration and washed with 5 °C distilled water (2 × 25 mL) and then methanol (5 × 15 ml). The crude product was then recrystallised by dissolving the compound in distilled water at 90 °C and leaving to cool to room temperature overnight. The purified product was collected by vacuum filtration and dried under reduced pressure. ^{1}H NMR (200 MHz, D_2O) δ (ppm): 4.27 (s, 2H), 3.21 (m, 2H), 2.13 (m, 2H), 1.73 (m, 2H), 1.38 (m, 4H).

2.2. R,R-1,2-diaminocyclohexane

(*R,R*)-1,2-diaminocyclohexane tartrate salt (13.2 g, 50 mmol) was dissolved in 40 ml distilled water and 50 mL CH_2Cl_2. To this, a cooled solution of sodium hydroxide (4.9 g in 40 mL distilled water) was added dropwise. Sodium chloride was added (3 g, 51.5 mmol), and then the reaction mixture was stirred for 30 minutes. Following this the aqueous phase was decanted and extracted with chloroform (3 × 20 mL), with the combined organic layers being dried over magnesium sulphate. The mixture was filtered and the solvent removed by rotary evaporation. The product was dried under reduced pressure and stored at -20 °C. ^{1}H NMR (200 MHz, $CDCl_3$) δ (ppm): 2.19 (m, 2H), 1.75 (m, 2H), 1.60 (m, 2H), 1.19 (m, 2H), 1.04 (m, 2H).

2.3. 5-chloromethyl-3-methoxysalicylaldehyde (1)

A mixture of 9 g (300 mmol) of para formaldehyde, 6g (44 mmol) of anhydrous zinc chloride and conc. hydrochloric acid (100 mL) into 500 mL two-necked RB flask was strongly stirred at room temperature under HCl atmosphere for 30 min, until complete dissolution of solids. After which a solution of 30 g (197.2 mmol) *o*-vanillin in 150 mL of benzene was added dropwise with continuous stirring under HCl atmosphere. Continue passing HCl gas over the vigorously stirred reaction mixture for further 4 h while the reaction temperature must kept below 20 °C. Then 100 mL and 100 g of crushed ice were simultaneously added to the black-red reaction mixture. A phase separation was observed from the mixture, and two layers were obtained. The benzene layer was separated by aspirating freed from resinous products, and thoroughly washed with deionized water until the pH of washing effluent reached 7. After drying over anhydrous sodium sulfate, the benzene was distilled off.

The obtained crude viscous brown-black oil was subjected to crystallization twice from petroleum ether 100-120 to furnish 5-chloromethyl-3-methoxysalicylaldehyde (**1**) (22.15 g, 56 %) as orange needles. FTIR (KBr, cm-1): 3437 (m, br, *v*(O-H)), 3086 (m, br, *v*asym(C-H), Ar), 3043 (m, br, *v*sym(C-H), Ar), 2970 (m, sh, *v*asym(CH2)), 2867 (m, sh, *v*sym(CH2)), 1645 (vs, sh, *v*(C=O)), 1451, 1395 (s, sh, *v*(C=CAr+ C-Hbend)), 1275 (s, sh, *v*(Ar-O)), 689 (s, sh, *v*(C–Cl)). 1H NMR (200 MHz, CDCl3) δ (ppm): 11.20 (s, 1 H, Ar-O**H**), 9.94 (s, 1 H, Ar-**H**C=O), , 7.35-7.11 (m, 2 H, 2 x Ar-**H**), 4.62 (s, 2 H, C**H2**-Ar), 3.98 (s, 3 H, OC**H3**). 13C NMR (126 MHz, CDCl3) δ (ppm): 196.56, 152.31, 149.14, 124.47, 120.58, 118.33, 118.27, 56.81, 56.72, and 46.21. EI-MS [C9H9ClO3] Calcd.: 200.62 Found: 200.00.

2.4. Synthesis of vanillyl imidazolium ionic liquids (2a,b)

To a vigorously stirred solution of 1-^{n}butylimidazole (2.25 g, 23.39 mmol) in dry toluene (10 mL) at room temperature was added the solution of 5-chloromethyl-3-methoxysalicylaldehyde (**1**) (4.15 g, 19.50 mmol) in dry toluene (10 mL), drop-wise over 30 min, under nitrogen atmosphere. The resulting solution was stirred under nitrogen atmosphere at 60 °C for 24 h. After cooling, the isolated product (**2a**) was washed intensively with 2 x 5 mL dry toluene, several with ether (5x10 mL), to remove the unreacted materials, and dried under vacuum to give the desired product which used for the following preparations without further purification. It was isolated as a yellowish-white solid, (89%); mp 54-55 °C. FT-IR (KBr, cm^{-1}): 3439 (m, br), 3051 (m, sh), 2958 (m, sh), 1649 (vs, sh), 1537, 1464, 1401 (s, sh), 1275 (s, sh), 1153 (s, sh). ^{1}H NMR (200 MHz, $CDCl_3$) δ (ppm): 10.68 (s, 1 H), 10.38 (s, 1 H), 8.95 (s, 1H), 7.78 (d, *J* = 1.98 Hz, 1 H), 7.70 (d, *J* = 2.00 Hz, 1 H), 7.31 (s, br, 2 H), 5.97 (s, 2 H), 4.12 (t, *J* = 7.0 Hz, 2 H), 3.87 (s, 3H), 1.76 (m_5, 2 H), 1.39 (m_6, 2 H), 0.92 (t, *J* = 7.1 Hz, 3H). ^{13}C NMR (125 MHz, $CDCl_3$) δ (ppm): 191.76, 158.04, 138.90, 137.76, 137.03, 130.21, 129.12, 127.97, 123.69, 122.89, 57.02, 48.55, 33.49, 22.79, 20.82, and 13.98. ESI MS: In positive mode peaks at m/z 287.2 a.m.u. ($[C_{16}H_{21}N_2O_2]^+$, $[M - Cl^-]^+$)

Anion metathesis: synthesis of (2b):

To a solution of vanillyl-butylimidazolium chloride (**2**a) (3.8 g, 11.75 mmol) in milli-Q water (50 mL) was added aqueous solution of solid $NaBF_4$ (1.37 g, 12.43 mmol) portion-wise with vigorous stirring while cooling in ice bath over 1 h. After the addition was completed, the reaction was stirred at room temperature for 24 h. The solid product was filtered, washed with milli-Q water (to remove $NaBF_4$ or HPF_6- solution and any water-soluble impurities) until it was neutral. The final product was dried under vacuum at 40 °C for 24 h. Samples of the isolated products are fully characterized below.

vanillyl-butylimidazolium tetrafluoroborate (2b): Yield (91%); mp 61-63 °C. FTIR (KBr, cm^{-1}): 3425 (m, br), 3095 (m, sh), 2968 (m, sh), 1649 (vs, sh), 1544, 1463, 1392 (s, sh), 1271 (s, sh), 1156 (s, sh), 1063 (vs, sh). ^{1}H NMR (200 MHz, DMSO-d_6) δ (ppm): 10.34 (s, 1 H), 9.22 (s, 1 H), 8.91 (s, 1H), 7.77 (d, *J* = 1.9 Hz, 1 H), 7.69 (d, *J* = 2.0 Hz, 1 H), 7.28 (s, 1 H), 7.26 (s, 1 H), 5.93 (s, 2 H), 4.17 (t, *J* = 7.1 Hz, 2 H), 2.66 (q, J = 7.0 Hz, 2H), 1.71 (m_5, 2 H), 1.36 (m_6, 2 H), 1.26 (t, J = 7.0 Hz, 3 H), 0.89 (t, *J* =

7.1 Hz, 3H). ^{13}C NMR (125 MHz, DMSO-d_6) δ (ppm): 192.12, 158.30, 138.83, 137.59, 137.21, 130.11, 128.89, 128.01, 123.70, 122.68, 56.99, 48.32, 33.16, 22.94, 20.85, 14.90 and 13.92. ^{19}F NMR (470 MHz, DMSO-d_6): -148.69 ppm (singlet). ESI MS: In positive mode peaks at m/z 287.2 a.m.u. ($[C_{17}H_{23}N_2O_2]^+$, $[M - BF_4^-]^+$)].

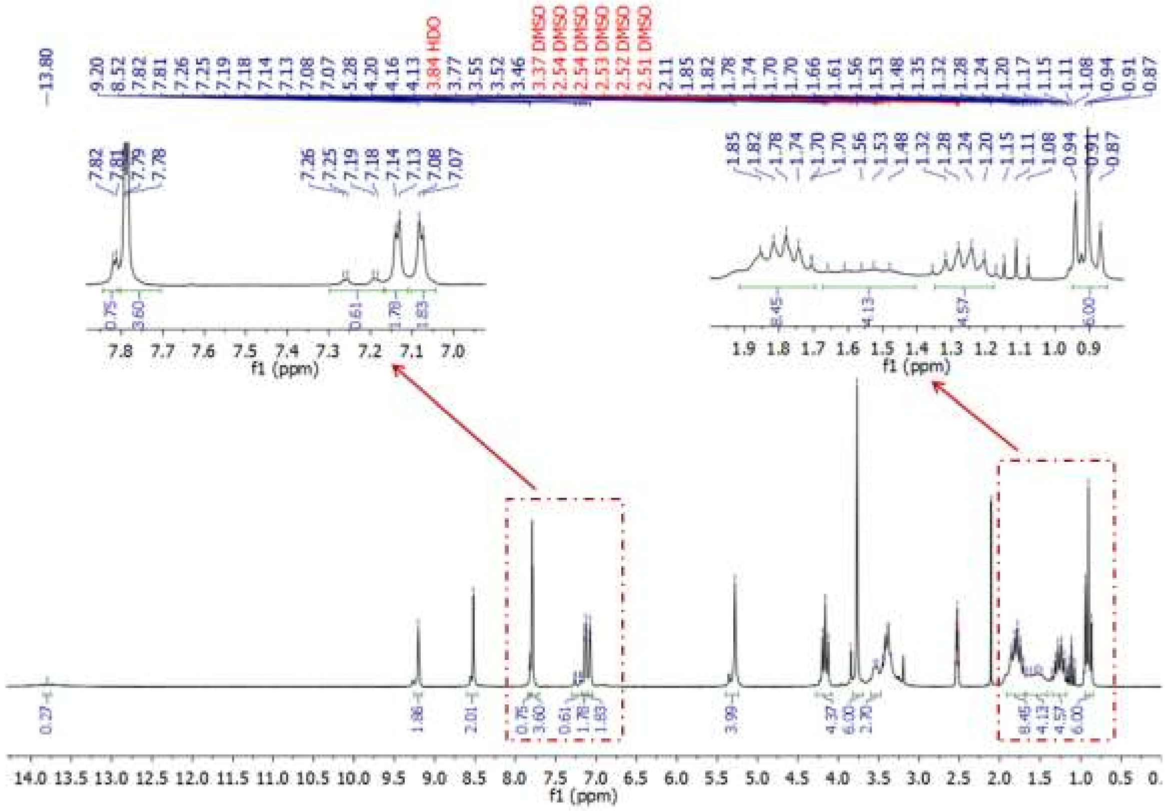

Figure S1: ^{1}HNMR spectrum of imidazolium-vanillylidene ligand (**H_2L_1**) (200 MHz, DMSO-d_6).

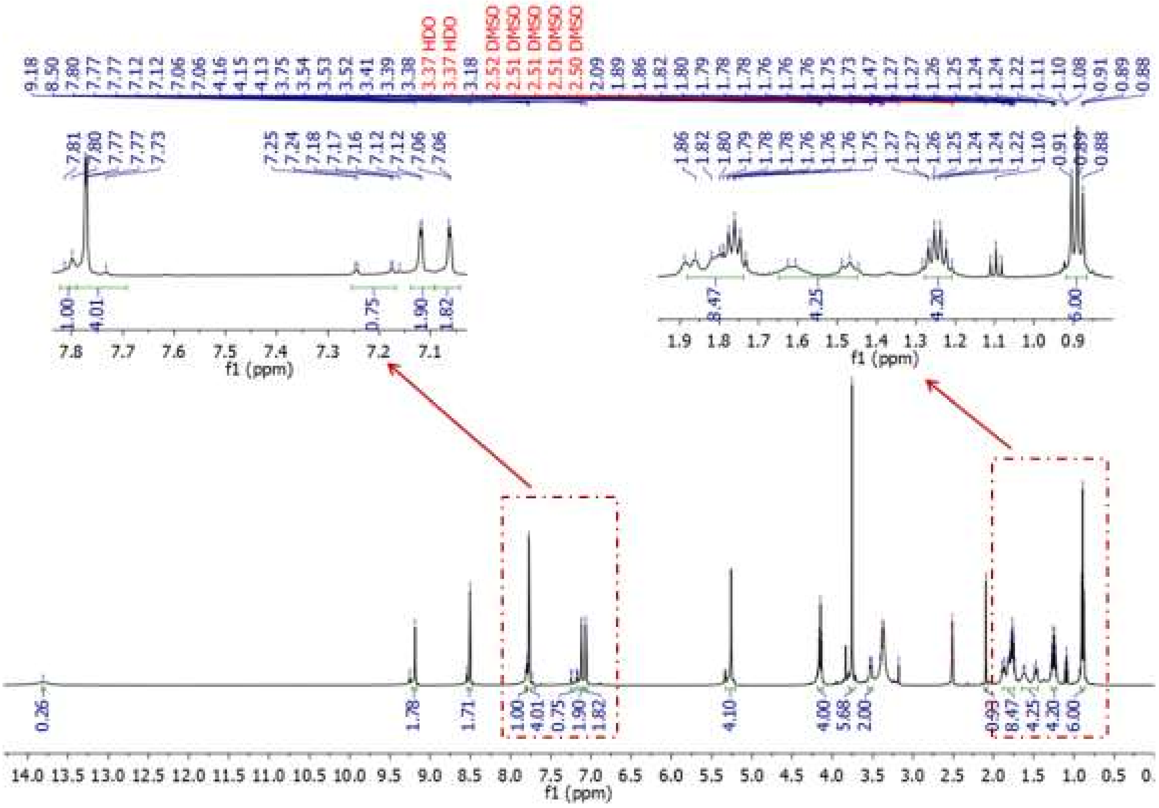

Figure S2: ^{1}HNMR spectrum of imidazolium-vanillylidene ligand (**H_2L_2**) (200 MHz, DMSO-d_6).

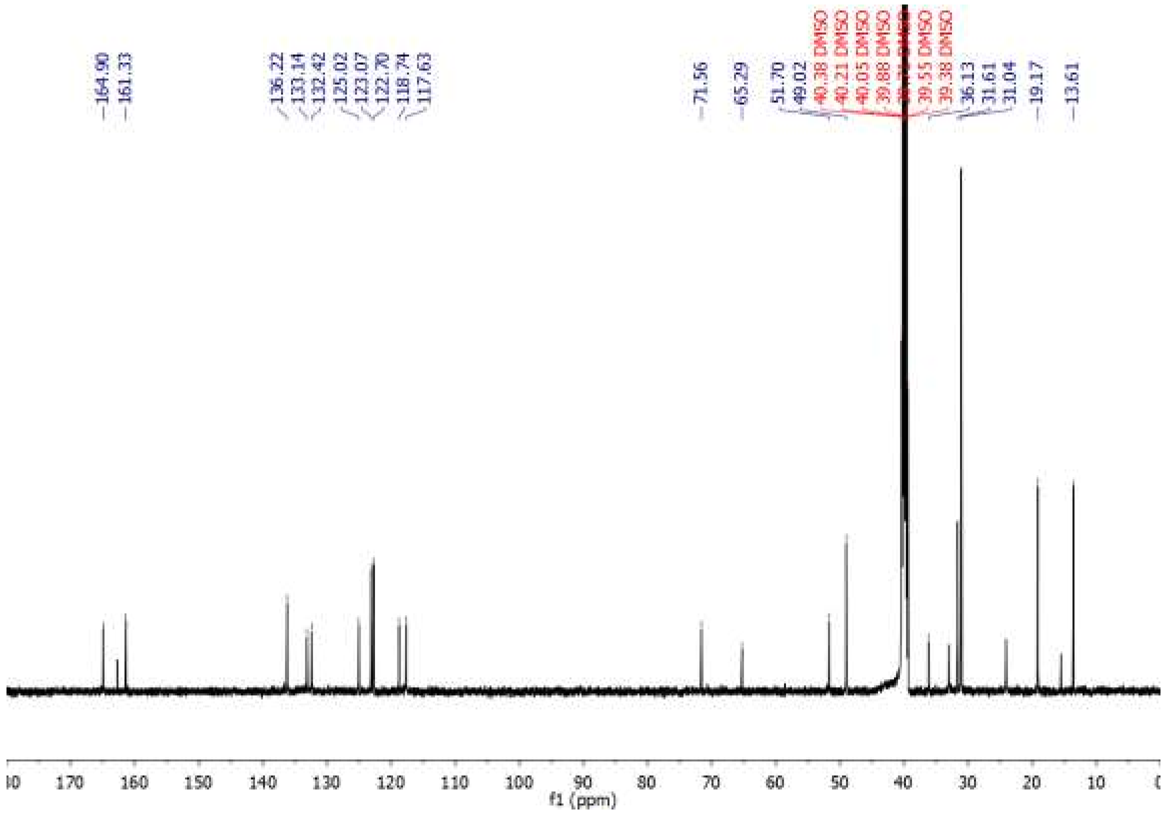

Figure S3: ^{13}C NMR spectrum of imidazolium-vanillylidene ligand (**H_2L_1**) (125 MHz, DMSO-d_6).

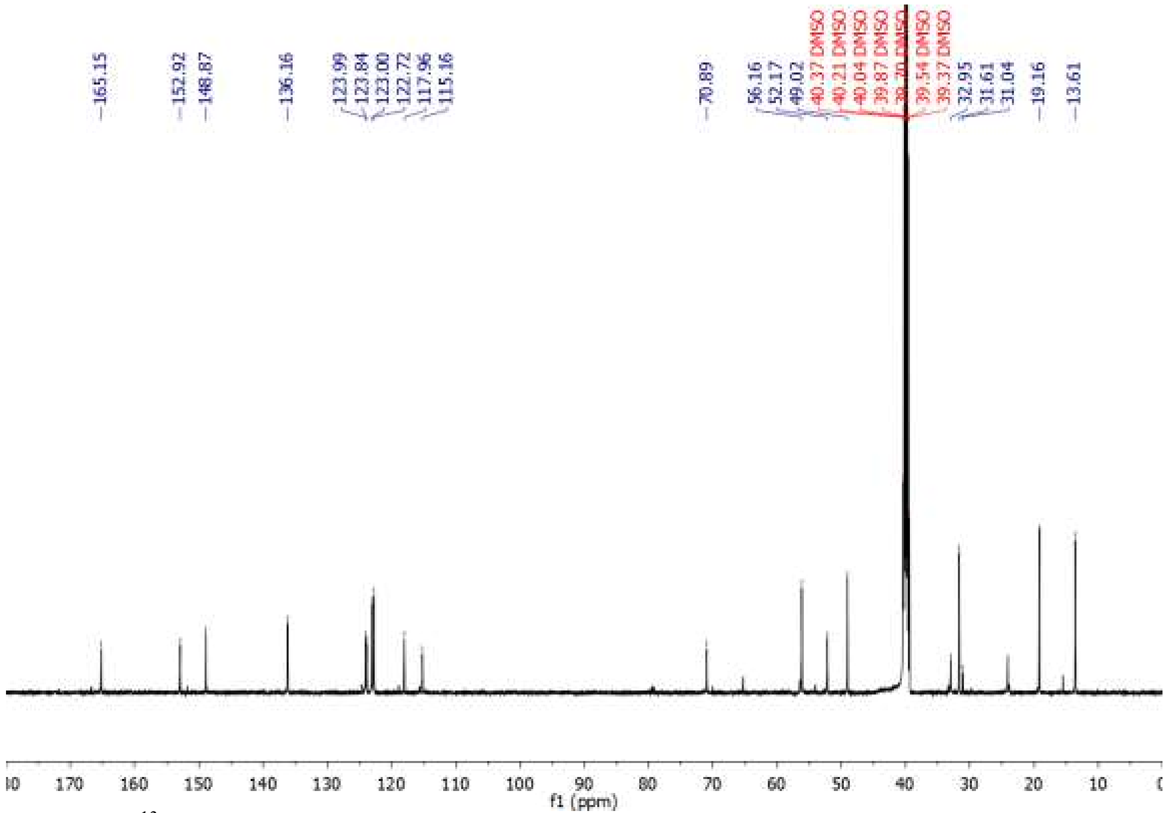

Figure S4: ^{13}C NMR spectrum of imidazolium-vanillylidene ligand (**H_2L_2**) (125 MHz, DMSO-d_6).

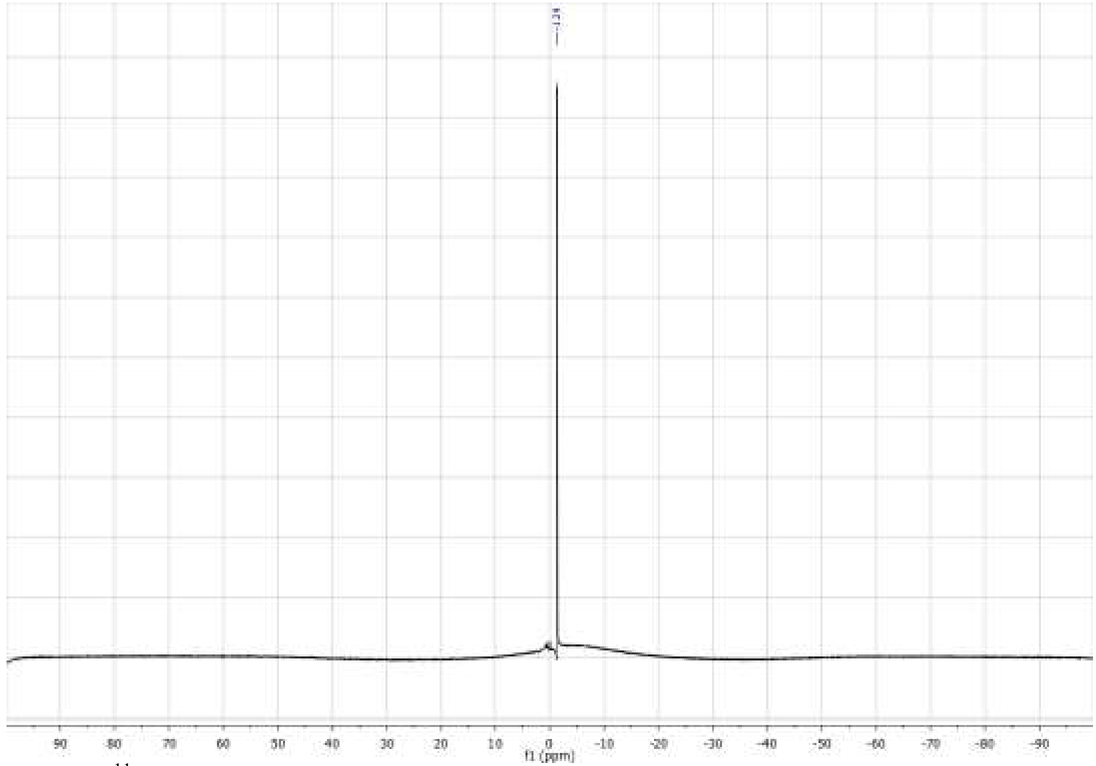

Figure S5: ^{11}B NMR spectrum of imidazolium-vanillylidene ligand (**H_2L_2**) (96 MHz, DMSO-d_6).

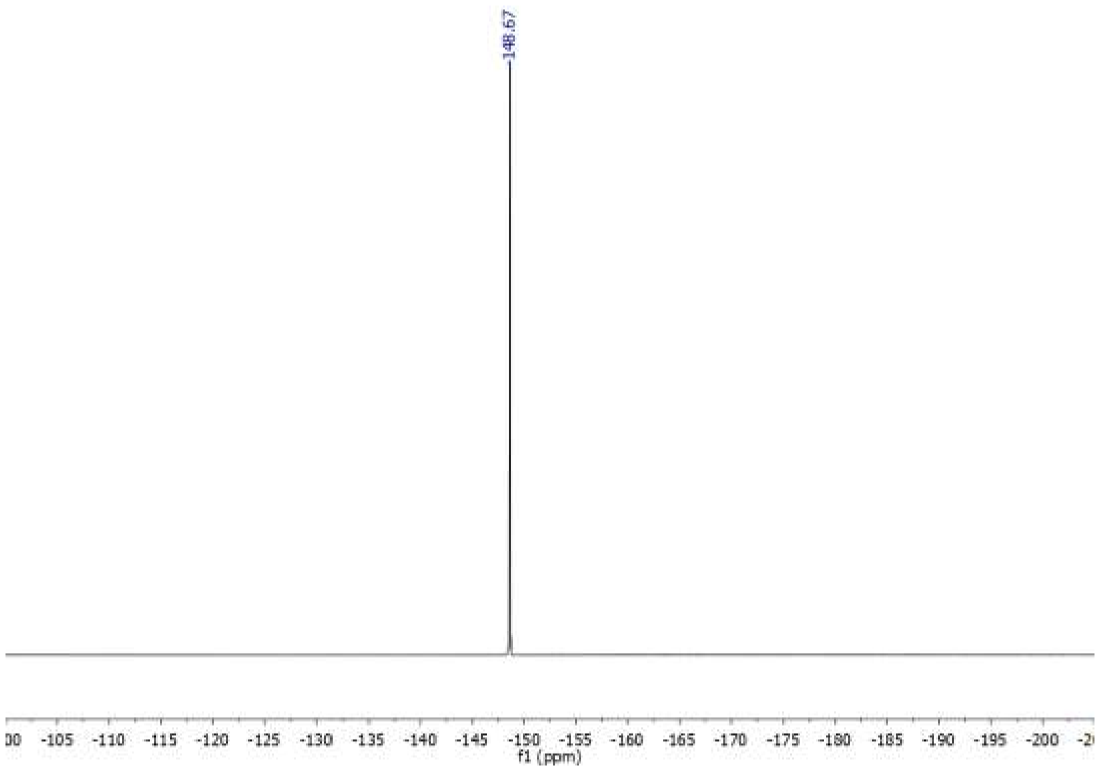

Figure S6: ^{19}F NMR spectrum of imidazolium-vanillylidene ligand (**H_2L_2**) (470 MHz, DMSO-d_6).

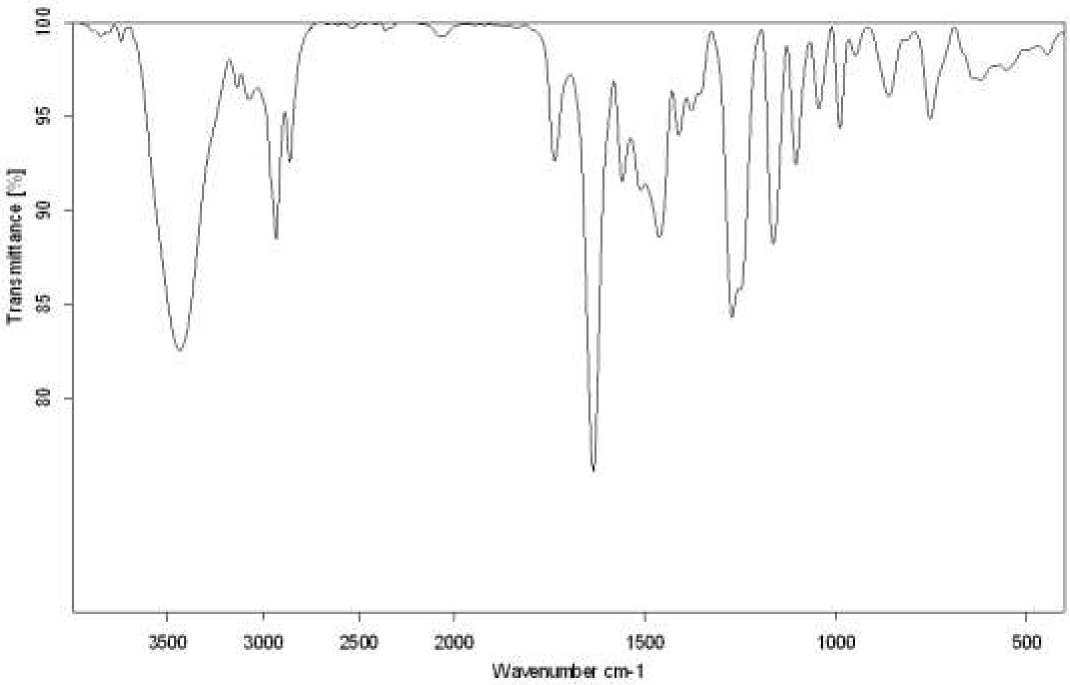

Figure S7: FTIR spectrum of imidazolium-vanillylidene ligand (**H_2L_1**)

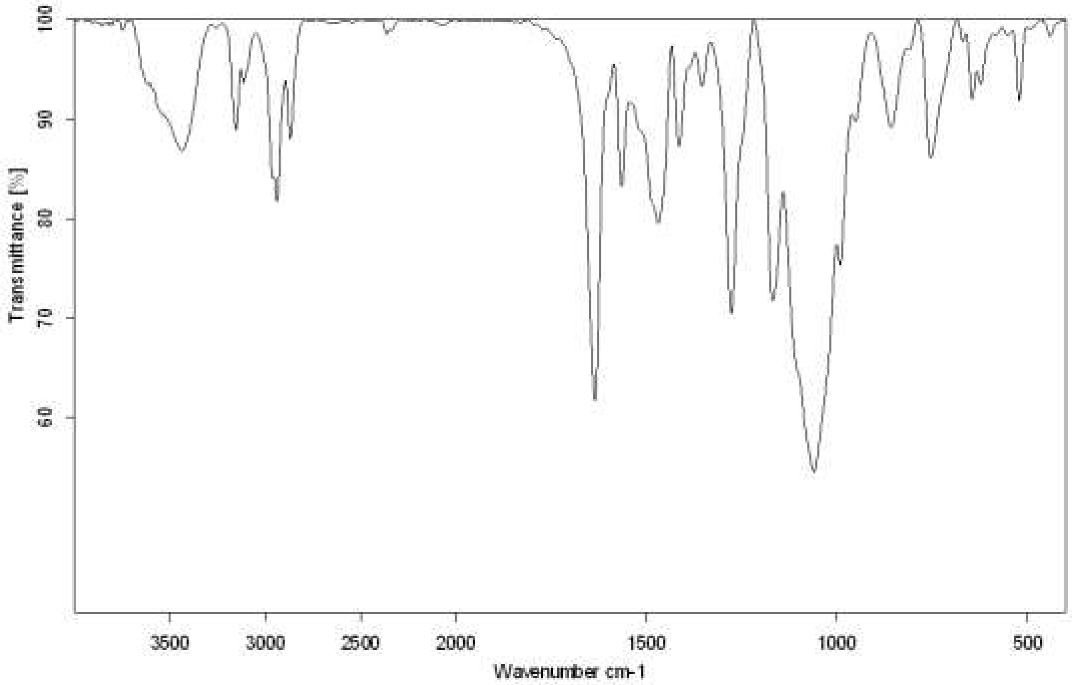

Figure S8: FTIR spectrum of imidazolium-vanillylidene ligand (**H_2L_2**)

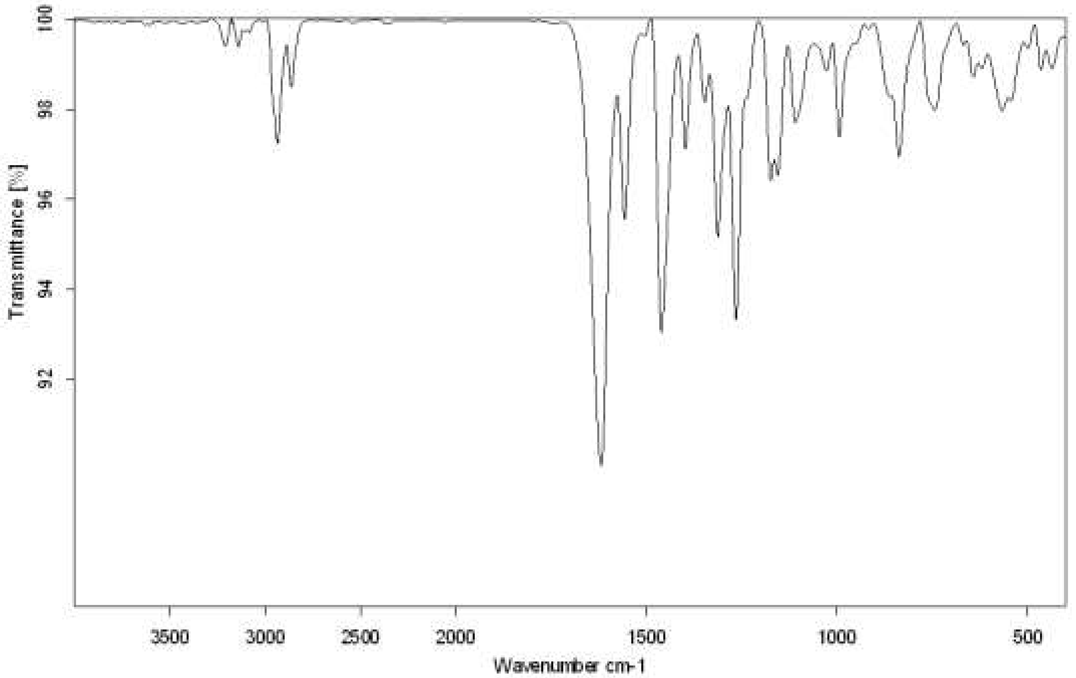

Figure S9: FTIR spectrum of Pd(II) imidazolium-vanillylidene complex (**PdL_1**)

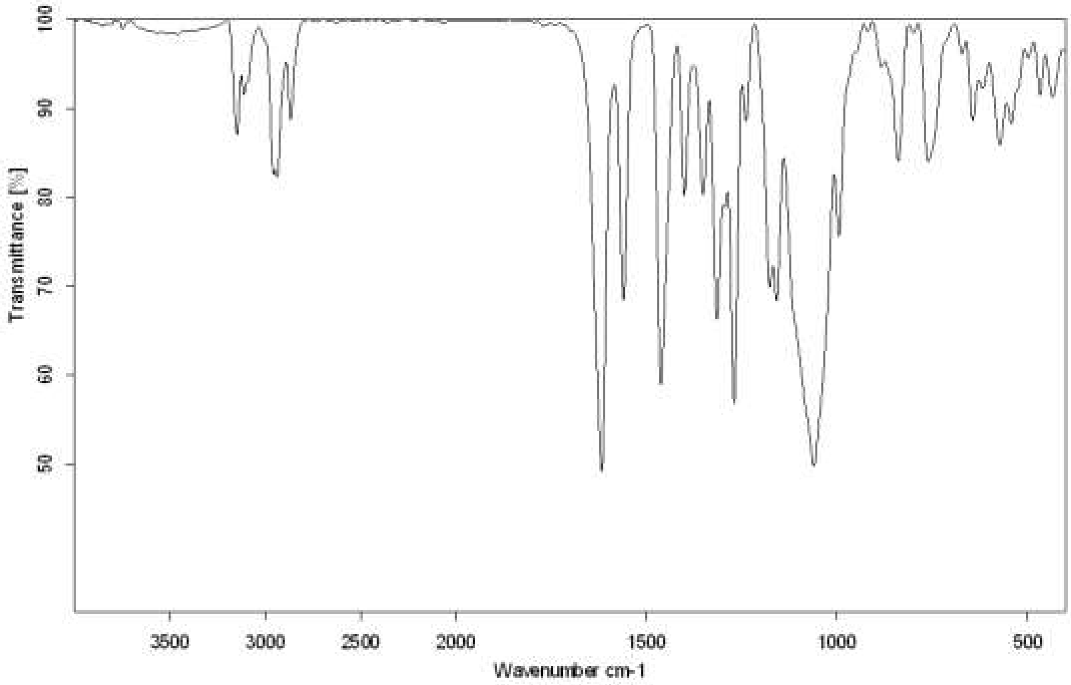

Figure S10: FTIR spectrum of Pd(II) imidazolium-vanillylidene complex (**PdL_2**)
